# Microbial and Genetic Resources for Cobalamin (Vitamin B12) Biosynthesis: From Ecosystems to Industrial Biotechnology

**DOI:** 10.3390/ijms22094522

**Published:** 2021-04-26

**Authors:** Larissa Balabanova, Liudmila Averianova, Maksim Marchenok, Oksana Son, Liudmila Tekutyeva

**Affiliations:** 1 Department of Bioeconomy and Food Security, School of Economics and Management, Far Eastern Federal University, 690922 Vladivostok, Russia; liudmila.tenkovskaia@gmail.com (L.A.); fury123j@gmail.com (M.M.); oksana_son@bk.ru (O.S.); tekuteva.la@dvfu.ru (L.T.); 2 Laboratory of Marine Biochemistry, G.B. Elyakov Pacific Institute of Bioorganic Chemistry, Far Eastern Branch, Russian Academy of Sciences, 690022 Vladivostok, Russia; 3 ARNIKA, Territory of PDA Nadezhdinskaya, 692481 Primorskiy Region, Russia

**Keywords:** coenzyme B12 family cofactors, biosynthesis pathways and regulation, genetic diversity, cobamide-producing strains, auxotrophy, cobalamin biotechnology

## Abstract

Many microbial producers of coenzyme B12 family cofactors together with their metabolically interdependent pathways are comprehensively studied and successfully used both in natural ecosystems dominated by auxotrophs, including bacteria and mammals, and in the safe industrial production of vitamin B12. Metabolic reconstruction for genomic and metagenomic data and functional genomics continue to mine the microbial and genetic resources for biosynthesis of the vital vitamin B12. Availability of metabolic engineering techniques and usage of affordable and renewable sources allowed improving bioprocess of vitamins, providing a positive impact on both economics and environment. The commercial production of vitamin B12 is mainly achieved through the use of the two major industrial strains, *Propionobacterium shermanii* and *Pseudomonas denitrificans*, that involves about 30 enzymatic steps in the biosynthesis of cobalamin and completely replaces chemical synthesis. However, there are still unresolved issues in cobalamin biosynthesis that need to be elucidated for future bioprocess improvements. In the present work, we review the current state of development and challenges for cobalamin (vitamin B12) biosynthesis, describing the major and novel prospective strains, and the studies of environmental factors and genetic tools effecting on the fermentation process are reported.

## 1. Introduction

Vitamin B12 is an important nutrient for humans and animals, which plays a key coenzyme role in numerous mitochondrial and cytosolic pathways (tricarboxylic acid cycle, one-carbon metabolism including methionine and folate cycles), methylation-mediated regulation (metabolites, DNA, RNA, and proteins), and regulation of sex steroids due to the host–microbe metabolic interactions, although it is essential for gut microbiota themselves [1,2]. It is widely used as a dietary supplement, as medicine for treating hematologic and neurological disorders, and as important feed additives (growth enhancer) for fowls and domestic animals. In addition, the B12-dependent bacteria that degrade steroids and chlorinated substanses in the environment due to agricultural and industrial impact are also important to preserve metabolic functions in mammals [2,3,4]. Vitamin B12 is related to compounds of the cobalt corrinoid group “cobalamins”, whose structure includes: cobalt-containing cyclic tetrapyrrolidine in the core (corrinoid ring); the common lower ligand 5,6-dimethylbenzimidazole (DMB) in the α-position; and one of four upper ligands in the β-position (cyano, hydroxyl, methyl or 5′-desoxyadenosyl radical) forming methylcobalamin (MeCbl), 5′-deoxyadenosylcobalamin (AdoCbl), hydroxocobalamin (OHCbl) and cyanocobalamin (CNCbl), respectively [5]. The natural forms of vitamin B12, MeCbl and AdoCbl, are synthesized only by prokaryotes (via aerobic/anaerobic and/or salvage pathways), which are required as essential cofactors for two enzymes: cytosolic methionine synthase (formation of methionine) and mitochondrial methylmalonyl-CoA mutase (formation of succinyl-CoA) in the human and animal metabolism [1]. In bacteria, the list of B12-dependent enzymes is complimented by glycerol dehydratase and ethanolamine ammonia lyase for anaerobic fermentation of glycerol, propanediols and ethanolamine; aminomutases for conversion of amino acids; ribonucleoside diphosphate reductases for DNA synthesis, and still growing [6,7]. Membrane proteins that are involved in the uptake of vitamin B12 are also yet to be identified [8]. In view of the instability of MeCbl and AdoCbl to the light, they are easily converted to OHCbl at room temperature in aqueous solution [9]. For this reason, almost all commercial vitamin B12 products (powder, tablets, capsules or granulas) having longer shelf life are produced as the air-stable cobalamin form CNCbl via a reaction with cyanide during industrial manufacture, which further is converted by animal and human organisms into the coenzymes MeCbl and AdoCbl [10]. Therefore, the term vitamin B12 is usually used to refer to CNCbl. However, the exogenous MeCbl and CNCbl were found to effect on the gut microbiome and microbial metabolism differently, although they have equal bioactivity in humans [11]. MeCbl reduced the diversity of gut microbiota, with stimulation of their lipid, terpenoid, and polyketide metabolism, as well as degradation of extracellular substances. Thus, the various forms of cobalamin in dietes should be still revised [11]. Meanwhile, the experimentally confirmed and bioinformatically predicted vitamin B12 requirements for growth (auxotrophy) have been shown for human gut bacteria, namely: *Ruminococcus bromi*, *Clostridium spiroforme*, *Serratia marcescens*, *Serratia fonticola*, *Shigella sonnei*, *Shigella flexneri*, *Shigella dysenteriae*, *Escherichia fergusonii*, *Escherichia coli*, *Lactobacillus sakei*, *Lactobacillus delbrueckii*, *Bacteroides thetaiotaomicron*, *Bacteroides ovatus*, *Bacteroides caccae* [12]. By metabolic reconstruction, the auxotrophy and capability of B12 biosynthesis were predicted in 60–80% and no more than 40% (Fusobacteria, Actinobacteria, Proteobacteria, Bacteroidetes, Firmicutes) of the human gut microbial genomes, respectively [12,13]. In the nature, the producers of vitamin B12 family cofactors (cobamides) are unevenly distributed across bacteria, which often metabolically coupled with algae, worms, plants and other organisms, with Actinobacteria enriched in and Bacteroidetes lacking in *de novo* biosynthesis (58 and 0.6%, respectively, among 11,000 publicly available genomes) [7]. Nevertheless, each of them, including possessors of the partial biosynthetic pathways, has a genomic potential for biosynthesis of cobalamins into an ecosystem [7,12,14,15,16,17,18,19,20]. Meanwhile, the uptake of exogenous vitamin B12 from environmental microflora is feasible not only in auxotrophic bacteria and mammals, but also in fungi and plants, probably, due to a nonspecific transport mechanism [7,12,21,22].

The industrial production of vitamin B12 can be achieved by chemical methods and bacterial fermentation processes. Nowadays, due to complexity of the chemical synthesis, which requires up to 70 steps, the commercially important vitamin B12 is manufactured by fermentation, using mutated and genetically engineered bacterial strains *P. shermanii* and *P. denitrificans*, with vitamin B12 yields up to 300 mg/L [5]. The production of cobalamin by these microorganisms has been extensively researched under specific culture conditions (supplementation of precursors and metal ions, carbon/nitrogen sources, oxygenic/anoxygenic conditions, cultivation time etc.), with consequence improving their synthesis capacity by random mutagenesis (UV light, chemicals) and genetic manipulations (overexpression, modification, regulation) [6,20,23]. In addition, the reported cobalamin-producing natural or recombinant bacteria comprised genera *Acetobacterium, Aerobacter, Agrobacterium, Alcaligenes, Arthrobacter, Azotobacter, Bacillus, Clostridium, Corynebacterium, Escherichia, Eubacterium, Flavobacterium, Methanobacillus, Methanosarcina, Mycobacterium, Propionibacterium, Proteus, Pseudomonas, Rhizobium, Rhodopseudomonas, Salmonella, Serratia, Streptococcus, Streptomyces, Xanthomonas* and others, whose habitats are soil, ocean, and microflora in digestive tracts of humans and animals [7,12,24,25]. A plenty of efforts for improvement of cobalamin biosynthesis were achieved through supplementation of the precursors 5-aminolevulinic acid (ALA) and DMB, and cobalt ions involved in catalysis, as well as by mutation and overexpression of the biosynthetic genes and/or riboswitch sequences in the selected strains [10,24,25,26]. However, the microbial production of vitamin B12 is still in very low yield due to the complexity of biosynthetic pathways that are required new solutions for the industrial strains’ improvement, and economically viable and efficient methods in the cobalamin biotechnology. Here, we describe the microbial vitamin B12 synthesis and variations of metabolic pathways, as well as their abundance and auxotrophy in various ecosystems, providing characteristics of strategies that were applied to enhancement of cobalamin production on the lab and industrial scale.

## 2. An Overview of Cobalamin Biosynthesis: Metabolic Pathways and Catalysts

**Figure 1:**
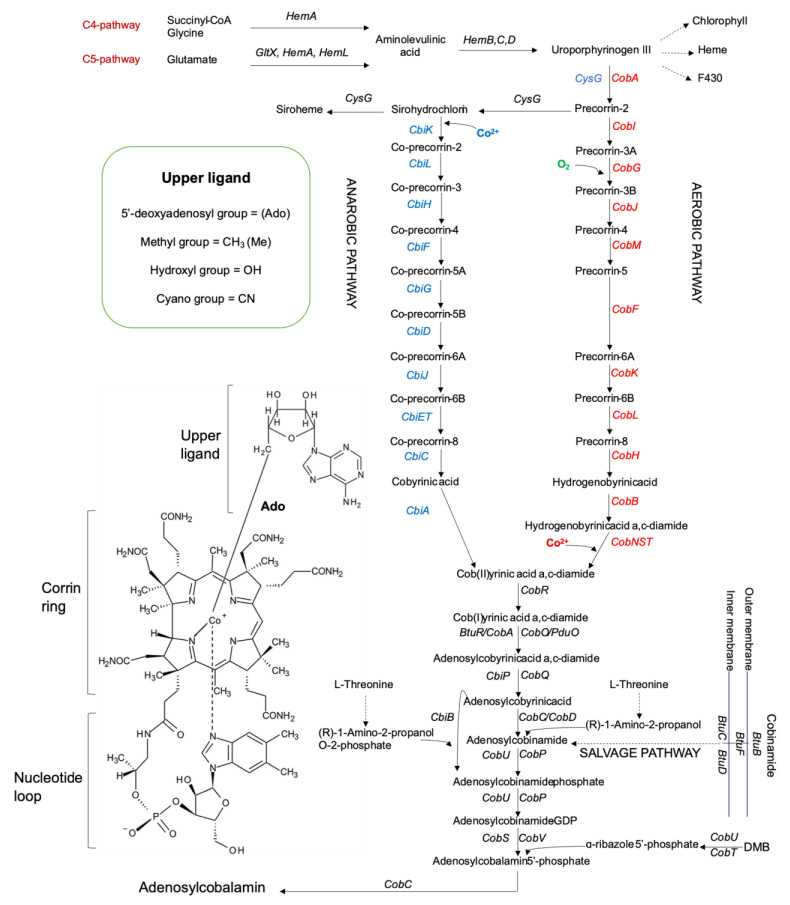
Scheme of the aerobic, anaerobic and salvage cobalamin biosynthetic pathways: the aerobic pathway is shown for *P. denitrificans* or *Sinorhizobium meliloti* (the intermediates are outlined on the right), and the anaerobic pathway and its intermediates are outlined on the left for well-studied bacteria *S. typhimurium*, *P. shermanii* and *B. megaterium*; the structure of vitamin B12 with the lower ligand 5,6-dimethylbenzimidazole (DMB) and the upper ligand consisting of either 5′-deoxyadenosyl, methyl, hydroxyl or cyan group, with their respective names being adenosylcobalamin (AdoCbl), methylcobalamin (MeCbl), hydroxocobalamin (OHCbl) and cyanocobalamin (CNCbl); the gene names are in blue, red, and black and used throughout this work. The anaerobic and aerobic cobalamin pathways are characterized by the early and late cobalt insertions, respectively. In the anaerobic pathway, CysG is a common enzyme for siroheme and cobalamin synthesis. Whilst decarboxylation of UroIII leads to the biosynthesis of hemes and chlorophylls, methylation of UroIII at C-2 and C-7 results in the synthesis of precorrin-2, that is also the last common intermediate in the synthesis of coenzyme F430 and siroheme [7,9,20,24].

Cobalamins (Cbl) are of the most structurally complex cofactors, which differ in their upper ligand (Figure 1). Microorganisms can produce cobalamin by *de novo* (aerobic/anaerobic) requiring up to 30 enzymatic steps or by contracted (salvage) pathways [6,7,12,19,20]. The *de novo* pathways include three majour stages: (1) production of uroporphyrinogen III (UroIII), the first macrocyclic intermediate in tetrapyrrole synthesis; (2) transformation of UroIII into cobinamide (Cbi) i.e., the corrin ring formation and adenylation; (3) nucleotide loop assembly i.e., synthesis of a lower axial ligand, usually 5,6-dimethylbenzimidazole (DMB), and the attachment to the corrin ring. The genes/enzymes of the oxygen-dependent and oxygen-independent pathways are designated as *cob*/Cob and *cbi*/Cbi, respectively (Figure 1). The major differences between these two pathways are the requirement for molecular oxygen in the assistance to ring contraction and cobalt insertion: only after the synthesis of hydrogenobyrinic acid *a*, *c*-diamide (late stage) in the aerobic pathway or at the precorrin-2 stage (early insertion) in the anaerobic pathway, and the biosynthesis of a lower ligand (usually DMB) [6,10,24,27,28]. Many intermediates and precursors of the both pathways are substrates/products of the similar enzymes (homologues or orthologues), but some of them are pathway-specific. The aerobic pathway was found in *P. denitrificans*, a well-known industrial producer of the coenzyme B12, of which gene clusters II and I were detailed characterized [26]. The anaerobic biosynthetic pathway was found in the anaerobic *Salmonella enterica* serovar Typhymurium, *P. shermanii* and aerobic *Bacillus megaterium* [24,29]. Figure 1 illustrates the biosynthetic pathways for *P. denitrificans* and *S. typhymurium*. Salvage pathways are achieved via absorbing exogenous Cbi by some bacteria from the environment, which later are mostly converted to AdoCbl by different enzymes [24]. Cbi has an incomplete corrinoid structure, which lacks the nucleotide loop and upper ligand [20].

### 2.1. Molecular-Genetic Organization of Cobalamin Biosynthetic Pathways

#### 2.1.1. Historical Implication of Cobalamin Biosynthesis Signatures

Biosynthetic pathways for complex tetrapyrrolic cofactors are combined in “Porphyrin and chlorophyll metabolism” by the KEGG database [30] and distinguished by the tetrapyrrole-derived framework with a central chelated metal ion (cobalt, magnesium, iron, or nickel) [20]. The complete and partial Cbl biosynthetic gene clusters, which contain *hem-cob* (aerobic) and *hem-cbi-pdu-etu* (anaerobic) operons, have been identified for a lot of available prokaryotic genomes by combination of genetic, biochemical and bioinformatic approaches, such as metabolic reconstruction and function prediction based on the comparative analyses of genes, operons, and regulatory elements [7,12,31,32,33]. They include the genes *hemALBCD* and *gltX* (EC 6.1.1.17, synthesis of L-glutamyl-tRNA or glutamic acid from glutamate) encoding for the homonymous enzymes for the synthesis of UroIII, which is the common intermediate for the heme, chlorophyll, and coenzyme F_430_ biosynthesis pathways [20].

The UroIII C-methyltransferase/siroheme synthase is encoded by *cobA* (aerobic bacteria) and multifunctional *cysG* (anaerobic bacteria) for the UroIII methylation at C2 and C7 [7,20]. The aerobic Cbl pathway includes five S-adenosyl-L-methionine (SAM)-dependent methyltransferases, CobA, I, J, M, and F, for introduction of six methyl groups and is characterized by the incorporation of molecular oxygen by a monooxygenase CobG. The anaerobic pathway also requires five methyltransferases CysG, CbiK/X, and CbiLHF (Figure 1). The cobalt chelation is catalyzed by the ATP-dependent heterotrimeric enzyme in the aerobic *P. denitrificans* consisting of the subnit CobN-magnesium chelatase (pfam02514), the subnit CobS-cobalamin 5′-phosphate synthase (TIGR01650), and the subunit CobT-cobalt chelatase (TIGR01651) or by the ATP-independent enzymes CbiK/X in the facultative anaerobes *S. typhimurium*/*B. megaterium* [31,34,35]. However, CobNST in the aerobic pathway are nongomologous to the enzymes with the same symbols in the anaerobic pathway due to their discovery history. Indeed, the enzymes CobT, CobU, and CobS had been first described for the nucleotide loop assembly in the anaerobic *S. typhimurium* and salvager *E. coli* (the partial Cbl biosynthesis from Cbi), before finding the aerobic nonhomologous cobalt chetalase complex CobNST in *P. denitrificans* [36]. Therefore, the *P. denitrificans* CobNST subunits are aligning rather with the structurally related enzymes, encoded by the nitrogen-fixing soil bacteria, such as *Mesorhizobium loti* and *Caulobacter vibrioides* (https://www.ncbi.nlm.nih.gov/Structure/cdd/TIGR01651, accessed on 15 March 2021). The *de novo* Cbl anaerobic pathway is considered as the classical variant 1 for genetic functional annotations that is prevalent among bacteria [7,12,20,31,37]. Meanwhile, the *de novo* Cbl aerobic pathway is designated as the variant 2 that is largely restricted to the members of Proteobacteria [7].

#### 2.1.2. Aerobic Pathway and Related Enzymes

Thus, the enzymes and their encoding genes (metabolic pathway signatures) for the corrin ring biosynthesis are nonortologous for these two pathways beginning from the precorrin-2 stage (Figure 1). The following signatures have been ultimately accepted for the aerobic pathway enzymes: (1) SAM-dependent bismethyltransferase CobA (no EC number) for methylation of UroIII at C2 and 7; (2) dimeric precorrin-2 C20-methyltransferase [CobI]_2_ (EC 2.1.1.130) for methylation precorrin-2 at C20; (3) precorrin-3B synthase CobG (EC 1.14.13.83) for installation of oxygen-derived functionality at C-20 for future ring contraction; (4) SAM-dependent bifunctional precorrin-3B C17-methyltransferase CobJ (EC 2.1.1.131) for ring contraction initiating methylation at C17; (5) SAM-dependent precorrin-4 C11-methyltransferase CobM (EC 2.1.1.133) for methylation at C11; (6) SAM-dependent precorrin-5 (C1)-methyltransferase or precorrin 6A synthase CobF (EC 2.1.1.152) for methylation and deacetylation of precorrin-5; (7) HADPH-dependent precorrin-6A reductase CobK (EC 1.3.1.54) for the reduction of the double bond between C-18 and C-19 of precorrin-6A; (8) multisubunit and multifunctional SAM-dependent precorrin-6B C5,15-methyltransferase [CobL]_8_ (EC 2.1.1.132) for methylation at C5 and 15, decarboxylation of the acetic acid side chain at position C12 of precorrin-6B to yield precorrin-8x; (9) dimeric precorrin-8X methylmutase [CobH]_2_ (EC 5.4.99.61) for methyl-migration from C11 to C12 position to convert precorrin-8x into hydrogenobyrinate; (10) dimeric cobyrinic acid *a,c*-diamide synthase [CobB]_2_ (EC 6.3.5.9) for amidation of cobyrinate and hydrogenobyrinate, with ATP and glutamine or ammonia, at *a* and *c* positions to transform them into cob(II)yrinate *a,c*-diamide and hydrogenobyrinate *a,c*-diamide, respectively, via the intermediate formation of *c*-monoamide form [7,20,29,38]. After cobalt insertion by the class I chelatase of multiple domains CobNST (EC 6.6.1.2), the reduction Co (II) to Co (I) is catalyzed by the flavin-dependent cob(II)yrinic acid *a,c*-diamide reductase CobR (no EC number) that results in the interaction with the corrinid adenosyltransferase CobO (EC 2.5.1.17) before adenosylation (Figure 1). However, the CobR-like reductases involved in the Cbl biosynthesis may be a non-specific flavoenzyme, as in *Brucella melitensis* due to the reduction of cobalt Co(I) predominantly by flavin [29].

#### 2.1.3. Anaerobic Pathway and Related Enzymes

For the anaerobic pathway, after the methylation of UroIII under the action of mostly often multifunctional C-methytransferases [CysG]_2_ (*S. typhimurium*, *E. coli*), [CorA]_2_ (*Methanobacterium ivanovii*), MET1 (*Saccharomyces cerevisiae*) (EC 2.1.1.107) or SirABC (*B. megaterium*) (EC 4.99.1.3), the NAD-dependent precorrin-2 dehydrogenase or siroheme synthase (EC 1.3.1.76) catalyzes production of sirohydrochlorin (Factor II) from precorrin-2 and has also several enzyme commission synonyms: [SirC]_2_ for *B. megaterium*, [CysG]_2_ for *S. typhimurium* and *E. coli*, [MET8]_2_ for *S. cerevisiae* [20,29]. Sirohydrochlorin is used by the class II homodimeric chelatases [CbiK/X]_2_ (*S. typhimurium*/*B. megaterium*) (EC 4.99.1.3) for the cobalt insertion and generation of cobalt-factor II (or cobalt-precorrin-2) (Figure 1). Then, the SAM-dependent methylation is catalyzed by the follow enzymes (for *S. enterica* serovar Typhimurium by default below): multifunctional cobalt-sirohydrochlorin C20-methyltransferase CbiL (EC 2.1.1.151) for generation of cobalt-factor III (cobalt-precorrin-3); cobalt-precorrin-3 C17-methyltransferase CbiH (no EC number) for resulting in the ring-contracted intermediate, cobalt-precorrin-4; cobalt precorrin-4 C11-methyltransferase for the cobalt-precorrin-5A synthesis [20,29]. The cobalt-precorrin 5A hydrolase CbiG (EC 3.7.1.12) catalyzes hydrolysis of the ring A acetate δ-lactone with the loss of carbon at C20 and its attached methyl group in the form of acetaldehyde (contraction of the porphyrin-type tetrapyrrole ring and its conversion to a corrin ring), wchich is methylated at C1 by the cobalt-precorrin-6A synthase CbiD (EC 2.1.1.195) and dependent on the presence of the enzymes CbiA and CbiP of the later stage [20,29]. The NADH-dependent cobalt-precorrin-6A reductase CbiJ (EC 1.3.1.106) has sequence similarity with the aerobic CobK catalyzing the reduction of C18/C19 double bond of the tetrapyrrole “D” ring in the next intermediate, cobalt-precorrin-6B. Decarboxylation at C12 by cobalt-precorrin-6B C15-methyltransferase CbiT (EC 2.1.1.196) produces cobalt-precorrin-7, with its further methylation at C5 by cobalt-precorrin-7 C5-methyltransferase CbiE (EC 2.1.1.289) to synthesize cobyrinic acid and amidation of the *a* and *c* side chains of the macrocycle by cobyrinate *a,c*-diamide synthase CbiA (EC 6.3.5.11) [20,29].

#### 2.1.4. Common Pathway and Salvage

At the stage of forming adenosyl cobyrinic acid *a,c*-diamide, the aerobic and anaerobic pathways combine and continue to be fulfilled by structurally similar enzymes (Figure 1). In the AdoCbl biosynthesis, cobyrinic acid *a,c*-diamide may be adenosylated at the cobalt ion by three type of ATP:corrinoid adenosyltransferases (EC 2.5.1.17): (1) BtuR (formerly CobA) in *S. enterica,* which is orthologous to CobO (EC 2.5.1.17) in *P. denitrificans*; (2) PduO in the (S)-propan-diol degradation pathway, when AdoCbl is a required cofactor of propanediol dehydratases (EC 4.2.1.28), encoded by *pdu* operon, and (3) EutT (EC 2.5.1.M19) in the ethanolamine utilization, when AdoCbl is a required cofactor for ethanolamine ammonia-lyases (EC 4.3.1.7), encoded by *eut* operon in *S. enterica* [20,29]. all three types are not homologues, but rather a good example of convergent evolution of proteins that catalyze the same reaction [7,39]. Many members from the structural adenosyltransferase-like superfamily (IPR036451), particularly the mammalian PduO-type sequences (IPR029499), have been found to be bifunctional enzymes that catalyse both reduction of cob(II)alamin to cob(I)alamin and adenosylation of cob(I)alamin (BRENDA:EC2.5.1.17) [40].

The final steps in B12 biosynthesis include final amidation, and synthesis and attachment of the lower nucleotide loop that can also be used to salvage cobinamides (Figure 1). All eukaryotes and many prokaryotes, including those having *de novo* AdoCbl pathways, can synthesize AdoCbl from an exogenous cobinamide with the use of the same enzymes and certain proteins to transport it into the cell [7,12]. Therefore, the genes responsible for the nucleotide loop assembly in a microbial genome may be a part of a *de novo* biosynthesis pathway. Alternatively, there are salvagers in microbiota, mostly from the phylum Furmicutes [7,12]. They only take up an extracellular cobinamide (Cbi I salvage pathway), which has to be converted it into the real intermediate, the last precursor AdoCbi-phosphate, assemble the nucleotide loop and attach the lower ligand (Figure 1). Both *de novo* and salvage pathways are often themselves of lacking some genes or having alternative unknown genes and their uncertain organization in a chromosome [7,12,34,35]. The partial biosynthetic pathways predominantly lacking the first step, with the potential to salvage Cbi or tetrapyrrole precursors have been shown for Cbi-15.1%, ALA-0.3%, porphobilinogen (PBG)–0.6%, hydroxymethylbilane (HMB)–0.6%, UroIII–0.04%, precorrin-2–0.6%, where 78% salavgers of tetrapyrrole precursors are host-assosiated bacteria [7]. Among the human gut microorganisms, the calculated salvage potential are for Cbl–25.3%, Cbi-8.5%, cobyrinate (Ba)-1,4%, cobyrinate diamide (Cbr)-1.9%, including the salvage pathways for the Cbl producres with de novo aerobic (3.3%) and anaerobic (26%) biosynthesis [12]. This highlights the importance of Cbl and precursors salvaging for both nutrition dependent bacteria and coordination of gene expression patterns between host and microbiome [7,16,41].

The de novo AdoCbl biosynthesis and Cbi I salvage pathways include the follow common genes: *cobQ/cbiP* encoding for the dimeric glutamine-hydrolyzing adenosylcobyric acid synthase CobQ/CbiP, with glutamine amidotransferase domain (5′-deoxy-5′-adenosylcobyrinic-acid-*a,c*-diamide:L-glutamine amido-ligase, EC 6.3.5.10) that catalyzes the four-step amidation sequence from cobyrinic acid *a,c*-diamide into cobyric acid; *cobD/cbiB* encoding for the AdoCbi-phosphate synthase CobD/CbiB (EC 6.3.1.10), attaching of an aminopropanol linker to the free carboxylic acid [20,29]. The aminopropanol is derived from threonine by the action of either PduX (in *Salmonella*-like bacteria) or nonorthologous BluE(F) (in Rhodobacterales) encoding for a kinase (EC 2.7.1.177), resulting in threonine phosphorylation, which is decarboxylated by the threonine-phosphate decarboxylase CobC (EC 4.1.1.81) for formation of (*R*)-1-amino-2-propanol O-2-phosphate [7,35,42] (Figure 1).

In an alternative AdoCbl salvage pathway (Cbi II pathway), the archeal gene *cobZ* encoding for an AdoCbi hydrolase CobZ (EC 3.5.1.90) serves to convert AdoCbi to adenosylcobyrate, which is then transformed into AdoCbi-phosphate by the action of AdoCbi-phosphate synthase CbiB (EC 6.3.1.10) [29,36,43].

The genes *cobP/cobU/cobY* encode for the AdoCbi-phosphate guanylyltransferases [CobP]_2_ in the *P. denitrificans*-like or [CobU]_2_ in the *S. enterica*-like bacteria (EC 2.7.1.156), and nonhomologous CobY (EC 2.7.7.62) in arheabacteria, *Halobacterium salinarum*, *Methanopyrus kandleri*, *Methanosarcina mazei*, *Methanothermobacter thermautotrophicus*, *Rhodobacter sphaeroides* (AdoCbl salvage from Cbi II), possessing frequently both kinase and guanylyltransferase activities to catalyze two different reactions of phosphorylation of AdoCbi to AdoCbi-phosphate (for salvage pathway) and conversion of the latter to AdoCbi-GDP (for both *de novo* and salvage pathways) [29].

The lower ligand DMB biosynthesis is catalyzed by the aerobic 5,6-dimethylbenzimidazole synthase BluB (EC 1.13.11.79), which was detaily described for *S. meliloti*, by fragmentation and contraction of a bound reduced FMNH_2_ cofactor and cleavage of the ribityl tail (with formation of d-erythrose 4-phosphate) or by reduction of flavin to activate molecular oxygen for its own cannibalization [29]. Linking DMB to nicotinamide mononucleotide is catalyzed by a base-activating phosphoribosyltransferase CobU/T (EC 2.4.2.21) with generation of α-ribazole phosphate, displacing GDP in AdoCbi-GDP by the AdoCbl 5′-phosphate synthase CobV/CobS (EC 2.7.8.26). Then, phosphate is removed by the action of threonine-phosphate decarboxylase CobC/D (EC 4.1.1.81) to yield AdoCbl (Figure 1). In the archaea *Methanothrix soehngenii*, *Methanosarcina barkeri* and *M. mazei,* the function of AdoCba/α-ribazole phosphatase (EC 3.1.3.73) was predicted to be carried by the nonorthologous CobZ with the domain of EC 3.1.3.73 [29]. The genes from the archaeal Cbi II salvage pathway are found in many bacterial genomes, indicating their archaeal origin through horizontal gene transfer [29,36,43,44].

The MetaCyc Metabolic Pathway Database search for each enzyme provides the related biosynthetic reactions at the detail level for the facultative aerobes *P. denitrificans* and *Rhodobacter capsulatus* and the facultative anaerobes *B. megaterium* and *S. enterica* serovar Typhimurium [29]. The similar AdoCbl biosynthesis pathway variant 2 (aerobic) is in *B. melitensis* and *S. meliloti*. The AdoCbl biosynthesis variant I (anaerobic) are also actual for *Chlorobaculum tepidum*, *Leptospira interrogans*, *Methanocaldococcus jannaschii*, *Methanothermobacter thermautotrophicus*, *Propionibacterium freudenreichii*, *P. freudenreichii* subsp. *shermanii* [29].

#### 2.1.5. Selectivity of Lower Ligand Activation

The prokaryotic variants of complete corrinoids (cobamides, Cba) include nearly 20 different structures in dependence on the nature of lower nucleotide loop and the base, while the cobamide Cbl is preferable by eukaryotes [20,22]. Three classes of lower ligands include 16 known structures found in the natural Cba, namely: benzimidazoles (benzimidazole [Bza], 5-methylbenzimidazole [5-MeBza], 5,6-dimethylbenzimidazole [DMB], 5-hydroxybenzimidazole [5-OHBza], 5-methoxybenzimidazole [5-OMeBza], 5-methoxy-6-methylbenzimidazole [5-OMe-6-MeBza], naphthimidazole), purines (hypoxanthine, adenine [Ade], 2-methyladenine [2-MeAde], 2-methylmercaptoadenine [2-SMeAde], 2-methylsulfinyladenine [2-SOMeAde], 2-methylsulfonyladenine [2-SO_2_MeAde], guanine), and phenolics (phenol [Phe], *p*-cresol [Cre]) [45,46].

For the aerobic DMB biosynthesis, the *bluB* sequences are most abundant in the predicted Cba-producing bacteria, particularly in Proteobacteria, while this function may be encoded into the fusion sequences *bluB/cobT* in Actinobacteria [7,20,27]. The canonical DMB activation system through alpha-ribazole salvage pathway encoded by *cobU/T* is inherent for Firmicutes [35]. The *cobT* gene encoding for a nicotinate mononucleotide (NaMN): base phosphoribosyltransferase in *Lactobacillus reuteri* CRL 1098 activates DMB. Meanwhile, *Lactobacillus coryniformis* CRL 1001 does not have own *cobT* gene, but possesses the multicomponent signal transduction genes *cblT* and *cblS*, encoding for an α-ribasol transporter and a kinase protein, respectively [35]. Some *L. reuteri* and *S. enterica* enzymes CobT showed the adenine type activation that correlated with their structure [45]. The sequence variations in *cobU/T*-like homologues and orthologues, as well as lower ligand availability, were demonstrated to contribute to the substrate specificity of the base-activating phosphoribosyltransferases CobU/T (EC 2.4.2.21) and, consequently, Cba structural diversity [47]. Although *S. meliloti* produces cobalamin, *E. coli* produces [2-MeAde]Cba when provided with cobinamide, *Veillonella parvula* and *Sporomusa ovata* produce [Cre]Cba in the nature, each ortholog has distinct selectivity to the different suplemented ligands dependent on the growth conditions, with the preference for the native Cba by their cobamide-dependent enzymes [46]. Thus, Cre, Phe, and dimethylphenols (DMP) were the only compounds that function as cobamide lower ligands in the methanol-dependent growth of *S. ovata*, indicating the phenolyl Cba role in methyl transfer reactions [48,49]. Therefore, a higth concentration of other type lower ligand in the medium inhibits the growth of *S. ovata* due to the non-specific Cba synthesis [46,48]. However, the closely related molecular structure allows enhancing or modulating the specificity of CobU/T-like enzymes by mutation of the essential amino acid residues for the guided biosynthesis of Cba [47].

Incorporation of phenolic compounds into Cba is encoded by the *cobU/T*-like genes *arsAB* [7,42,46,47,48,49]. Probably, the ability of ArsAB to activate DMB or other benzimidazoles is a remnant of the evolution of an enzymatic activity that is of no use to *S.ovata*, either due to inability to synthesize DMB or the absence of this base in its environment [46]. Although phenolyl Cba are abundant in mixed microbial communities (16% in human feces, 20–34% in bovine rumen, and 70% in a trichloroethene-degrading enrichment community), only the acetogenic *S. ovata* DSM 2662 and human intestine *V. parvula* DSM 2008 are the only organisms known to produce phenolyl Cba [48,49]. *V.* parvula additionally has cobT that is differentially expressed with *arsAB* in order to produce varying ratios of [Cre]Cba and [Bza]Cba under different environmental conditions [48]. However, twenty-seven species belonging to the class Negativicutes in the phylum Firmicutes, such as anaerobic methophilic the termite gut-derived *Acetonema longum* and *Pelosinus propionicus* or soil and plant-derived *Anaeroarcus burkinensis* and *Anaeromusa acidaminophila*, respectively, putatively produce phenolyl Cba [7,50] (Table 1).

The purinyl Cba was found to serve as a native prosthetic group of reductive dehalogenases in organohalide-respiring bacteria, which provide a potential solution to remediate contaminated sites through the Cba-mediated dechlorination [3,4,32,33,45]. The CobT (*Dsf* CobT) in the strictly anaerobe *Desulfitobacterium metallireducens* strain DSM 15288 activated purine to its respective α-ribazole-5′-phosphate form. A phylogenetic clade of the Peptococcaceae CobT was distinct (<52% amino acid identity) from any other CobT implicated in phenol–*p*-cresol, adenine, guanine–hypoxanthine or Bza-type lower base activation [45].

For obligate anaerobes, DMB is formed from 5-aminoimidazole ribotide (thiamine and purine biosynthesis pathways) with participation of the genes *bzaABCDE/F* [20,29,51]. These genes were described for *Eubacterium limosum*, *Geobacter sulfurreducens*, *Moorella thermoacetica*, *Acetobacterium woodii* and suggested for their use in prediction of the different Cba structures with the different lower ligands, such as [5-OHBza]Cba (Factor III), [5-OMeBza]Cba (Factor III_m_), and 5-OMe-6-MeBza [20,29,51]. Thus, *G. sulfurreducens* pos-sesses the *bzaF* and *cobT* genes and produces [5-OHBza]Cba, *M. thermoacetica* has the *bzaA-bzaB-cobT-bzaC* operon and produces [5-OMeBza]Cba, and *E. limosum* and *A. woodii* contain the *bzaA-bzaB-cobT-bzaC-bzaD-bzaE* operons and produce Cbl [51].

## 3. Genetic Diversity of Cobalamin (Cobamide) Biosynthesis Pathways and Transport

### 3.1. Chromosomal Organization of Cobalamin Biosynthesis Pathways

**Figure 2:**
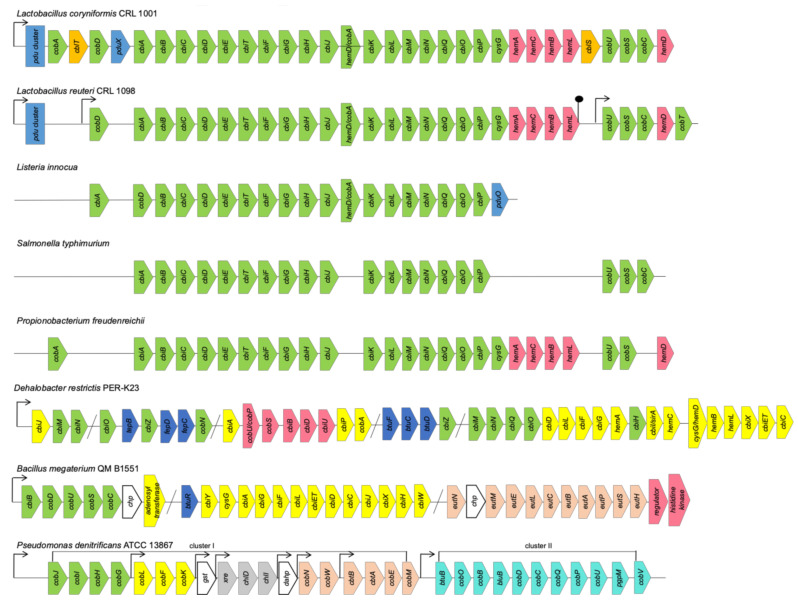
Genetic organization of B12 biosynthesis in different bacteria. The genes are indicated by colorful arrows; fine arrows denote confirmed promoters; hairpins symbols denote confirmed terminators [26,32,34,35,52,53].

In many cases, the coenzyme B12 biosynthetic pathways in the bacterial genomes distinguish from the canonical genetic organization of anaerobic (1) or aerobic (2) variants, as well as the Cbi I or Cbi II salvage pathways, by the number, order and combination of the operons and their genes’ content [7,12,44]. Frequently, even within a species, the Cbl gene clusters are characterized by missing one or several genes or their significant rearrangements in the operons, as well as along a chromosome (Figure 2).

#### 3.1.1. Clusters and Operons in Anaerobic Pathway

Thus, the *L. reuteri* Cbl biosynthetic cluster consists of two operons, with 29 open reading frames (ORFs), which translate two tandem transcripts carrying the sequences *cobD*, *cbiABCDETFGHJ*, *cobA/hemD*, *cbiKLMNQOP*, *sirA*, *hemACBL*, and *cobUSC*, *hemD*, *cobT*, respectively. The coding DNA sequences (CDSs) are similar to those coding for the anaerobic B12 pathway (variant 1) characterized for a few representatives of the genera *Listeria* and *Salmonella* (Figure 2). However, *L. reuteri* CRL 1098 and *L. coryniformis* CRL 1001 have different pathways for the last steps of the Cbl synthesis, at the stage of the lower ligand activation described above [35,53]. The amount of the genes organized into the clusters and involved in the Cbl biosynthesis of *Propionobacterium* are also strain-specific (Figure 2). Only two the biosynthesis clusters are in *P. acne* and *P. acidipropionici*, whereas in *P. freudenreichii* the Cbl biosynthesis genes are organized in four clusters encoding respectively for a cobalt transporter, anaerobic pathway, corrin ring modification and UroIII formation [54]. Most of such clusters can vary by the presence or absence of some transporters and fused genes [55]. The biotechnologically important *B. megaterium* strains QM B1551 and DSM319 have also two distinct independent operons: *cbiWHXJCDETLFGA-cysG-cbiYbtuR* and *cbiB-cobDUSC* for anaerobic and aerobic stages of B12 biosynthesis, respectively (Figure 2). The gene *cbiP* encoding for the adenosylcobyrinic acid synthase (EC 6.3.5.10) locates in their genomes as a single gene [52]. In addition, the genomes of both strains possess the B12-dependent gene locus, with the species-specific ethanolamine utilization operon *eutHSPABCLEM* together with an upstream ethanolamine two-component response regulator system and further uncharacterized genes contributing to ethanolamine utilization. The second *eut*-containing operon was found as a part of a small *eutABC-eat* cluster, where *eat* encodes an ethanolamine permease [52].

The CDSs related to the *pdu*- or *etu*-like genes and their regulatory proteins are commonly used to identify an anaerobic style of Cbl biosynthesis, which is intendent for gut microbiota (Figure 2). Furthermore, the coenzyme B12-dependent degradation of 1,2-propanediol (1,2-PD) by the enzymes from the bacterial cell’s specialized Pdu microcompartment (Pdu MCP) have been shown to protect bacteria from the toxic product propionaldehyde and involve them in an enteric pathogenesis [56]. About 50 genes organized in the *pdu* operons and their adjacent *cib* operons were induced by 1,2-PD, using a single regulatory protein PocR in *Salmonella, Shigella, Lactococcus, Lactobacillus, Yersinia, Listeria, and Klebsiella* [57]. The *pdu* genes locus consisting of 24 genes are organized in the operons: *pduABB’CDEGHJKLMNOPQSTUVWX*, with the PduMCP structural and degradative proteins-encoding genes *pduABB’JKMNTU* and *pduCDELPQW*, respectively, *pduF* encoding for 1,2-PD diffusion facilitator, and *pocR* encoding for transcriptional regulator. They are specifically involved in the anaerobic 1,2-PD utilization in the AdoCbl-dependent manner for which simultaneous *de novo* B12 synthesis is required [56,57]. Two global regulatory systems Crp/Cya and ArcA/ArcB affect inducibility of the *cib* and *pdu* operons. The *pocR* transcription is regulated by three promoters, which are controlled by both global regulatory proteins and autoinduction [57].

#### 3.1.2. Clusters and Operons in Aerobic Pathway

The aerobic (variant 2) Cbl biosynthetic clusters are well characterized for *P. denitrificans*, *S. meliloti*, *R. sphaeroides* and *Pseudomonas aeruginosa* [24,26,29,58]. The genome of *P. denitrificans* ATCC 13867 contains eight Cbl operons: seven in the cluster I and one in the cluster II (Figure 2). The genes *cobGHIJ*, *cobLFK*, and *cobM* involved in the initial steps of B12 biosynthesis, from precorrin-2 to precorrin-8, are located in the operons Opn1, Opn2 and Opn7, respectively [26]. In the operons Opn3, Opn4 and Opn5, the genes *gst* (unknown function), *chlI* and *chlD* (for magnesium chelation), *xre* and *dahp* (unknown function), respectively, are included (Figure 2). The genes *cobWN* and *cbtBA-cobEM*, located in the operons Opn6 and Opn7, are involved in cobalt chelation and transport, respectively. The single operon Opn8-containing cluster II encoding for the transcript *btuB-cobOB-bluB-cobDCPU-bgpM-cobV* is for fulfilment of the later Cbl biosynthetic steps, from hydrogenobyrinate to the coenzyme B12 [26].

#### 3.1.3. Transport Systems in Bacteria

Microorganisms may encode the partial biosynthetic pathways for converting one variant Cba to another apart from completing the biosynthesis from an intermediate (complete corrinoid salvaging) [59]. The TonB-dependent outer membrane corrinoid transporter, BtuB, has been showed to mediate the uptake of all major Cba variants, particularly such B12 family cofactors as cobalamin, pseudocobalamin and p-cresolylcobamide, and the intermediate Cbi [59]. BtuB is found only in Gram-negative bacteria, while the periplasmic binding protein BtuF and the ABC transporter BtuCD are found across bacterial taxa (Figure 1). The *B. thetaiotaomicron* genome contains three predicted B12 transport systems that are each located adjacent to a B12-riboswitch and each contains a BtuB homologue, with the functional specificity to the corrinoid structure that play distinct roles in the microbial fitness [60]. The BtuBFCD transporter exists in a single copy in *E. coli* and many other bacteria studied to date [44,60]. The multiple members of corrinoid transporters and salvaging enzymes besides the five distinct corrinoid biosynthesis-associated operons were found in one of the strains of organohalide-respiring bacteria *Dehalobacter restrictus* PER-K23 [34]. The strain PER-K23 contains a complete set of corrinoid biosynthetic genes. However, the gene *cbiH* was truncated and, therefore, nonfunctional that may explain the corrinoid auxotrophy of PER-K23 [34]. Recently, a novel structure of a transporter BtuM binding vitamin B12 in its base-off conformation, with a cysteine residue as axial ligand of the corrin cobalt ion, has been established in *Thiobacillus denitrificans*. BtuM supported B12-dependent bacterial growth and catalyzed decyanation of CNCbl [8]. However, the identification of transport systems, such as BtuFCD(B) of ATP-binding cassette (ABC) family (orthologues) and CbrT of the ECF family (paralogues), for a B12 pathway in genomes remain an open problem because of their involvement in the uptake of alternative B12 vitamers, such as thiazole, quinolinate, dethiobiotin, and pantoate [12].

### 3.2. Cobalamin Biosynthesis Capability Assessment in Bacteria

#### 3.2.1. Genetic Signatures of Cobalamin Pathways and Transport

Accordingly, the ratio of relative abundance of the genes *cob/cbi* responsible for the synthesis of Cbl-producing enzymes and the transporter-encoding gene *btuB* were suggested for a rapid assessment of Cbl production and transport potentiality in the environmental metagenomes [36]. Thus, it was found that in the soil microbiomes, the predominant Cbl producers were of Proteobacteria, Actinobacteria, Firmicutes, Nitrospirae, and Thaumarchaeota. However, a much larger proportion of the soil genera restricted themselves by the Cbl transport systems and DMB synthesis [36]. Metabolic subsystems of the genes involved in biosynthesis and salvage of Cbl (coenzyme B12) in the reference human gut bacterial genomes were identified by Rodionov et al. [12], with the use of the follow signatures for canonical anaerobic and aerobic pathways: *cbiLHFDGJTECA* and *cobGF*, respectively; for adenosylation *btuR/O* and downstream biosynthesis/salvage: *cbiPB, cobUSC, cblZ, pduX, cobDT*; for cobalt insertion of the *de novo* biosynthesis: *cbiKXX2* (anaerobic), *chlLD*, *cobN*(aerobic); and transporters for B12 uptake: *btuDFC*, *btuB*, *cbrUVT* (cobyrinate diamide uptake); for DMB uptake: *cblTS*; for cobalt uptake: *cbiMNQO*, *niCOT*, *hupE*, *cnoABDC*, *cbtFACDX* [12]. The Cba biosynthesis content in the genomes of different microbial producers was verified by the presence/absence of the similar sets of genetic signatures, including tetrapyrrole precursor biosynthesis *hemA,AL,BCD*, *cysG/cobA*; and *bluB*, the aerobic synthase for DMB, in the work of Shelton et al. [7]. Remarkably, the corrin ring biosynthesis markers, such as *cbiL*, *cbiF*, *cbiC* (anaerobic) and their ortologues *cobI*, *cobM* and *cobH* (aerobic), whose possessers are hightly abundant by nucleotid loop assembly annotations *cbiP/cobQ*, *cbiB/cobC/cobD*, *cobU/cobP*, *cobU/cobP/cobY*, are more predictive from the threshold-based Cba biosynthesis in the experimentally-verified Cba producers than the biosynthetic genes *cbiA/cobA*, which are found in 99% genomes of the predicted Cba producers [7]. The cobalt chelatase *cobNST* and *cbiX/K* annotations in genomes may be also nonspecific and belong to other metal chelatases in some bacteria that lack most of the corrin ring and nucleotide loop assembly genes [7].

However, there are still discovering new genes, metabolites or even pathways related to the Cbl-type corrinoid compounds production in dependence on the species and growth culture conditions (intermediaries) [7,35,51,60]. Thus, the sequence-based prediction of Cba were developed exclusively for only obligate anaerobic microorganisms, with the recently described structural genes *bzaABCDE/F* nessesary and sufficient for the anaerobic biosynthesis of DMB [7,51]. The *bza* operon from the anaerobic bacterium *M. thermoacetica* was shown to translate the hydroxybenzimidazole synthase BzaAB, phosphoribosyltransferase CobT, and the methyltransferases BzaC, BzaD and BzaE, that determine a new pathway for the regiospecific Cba biosynthesis and activation of the benzimidazolyl lower ligand [51]. Many prokaryotic membrane transporters involved in the uptake of vitamin B12 are yet to be identified [8,12]. The B12-based symbiotic relationships is often concomitant with the synthesis of a broad-spectrum or, in reverse, narrow-specific antimicrobial compounds for succeed colonization of surfaces and their microbiome species control, as it has been proved for the reuterin (3-hydroxypropionaldehyde) production in the B12-producing *Lactobacillus*. Therefore, their biosynthesis and other cross-related pathways may be also used for development of the method to determine the B12 family cofactor synthesis potentiality [12,35].

#### 3.2.2. Metabolic Reconstruction Methods

The whole-genome sequence-based metabolic reconstructions have been performed for the microbial species from soil, gut, marine environments, including algal and human microbial communities, to elucidate the mechanism of auxothrophy to amino acids and cofactors [33,60,61,62,63,64]. Genome functional annotations from Enzyme Commission (EC) numbers (http://www.sbcs.qmul.ac.uk/iubmb/enzyme/, accessed on 8 April 2021), the Pfam protein families database (http://pfam.xfam.org; Pfam 34.0 (accessed on 15 March 2021, 19,179 families), the TIGRFAM database of protein family definitions (http://tigrfams.jcvi.org/cgi-bin/index.cgi; Current Release: 15.0, 4488 families, accessed on 16 September 2014), Clusters of Orthologous Groups (COG) (https://www.ncbi.nlm.nih.gov/research/cog, accessed on 15 January 2021), The Integrated Microbial Genomes (IMG) system (https://img.jgi.doe.gov/, accessed on 9 April 2021) for Cba biosynthesis, Cba-dependent and -independent enzymes are used for identification of both Cba biosynthesis and Cba-dependence in genomes [7]. For the genes without a defined function (annotated as hypothetical genes) in a database, the experimentally characterized sequences are used as the query genes in BLASTP programs against the targeted genomes [7,12]. Rodionov and co-authors [12] applied in silico metabolic reconstructions based on the functional gene annotation in the extended SEED subsystems, using homology-based methods and three genome context techniques: clustering of the genes-signatures for the Cbl pathways into operons, co-regulation of genes by a common regulator or a riboswitch, and co-occurrence of genes in a set of related genomes. Based on the metabolic reconstruction of 2228 reference genomes from human gut microbiome (HGM), the representatives were classified into 10 groups according to the variants of the metabolic pathways: *de novo* anaerobic and aerobic Cbl biosynthesis or protothrophy (P1 and P2); *de novo* anaerobic and aerobic biosynthesis and salvage (P1&S and P2&S); cobyrinate auxotrophy and salvage (Aca&S); cobyrinate diamide auxotrophy and salvage (Acbr&S); cobinamide auxothrophy and salvage (Acbi&S); cobalamin/B12 auxothrophy and salvage (A&S). The species with the most numerous sequenced genomes from HGM (available in the GenBank, NCBI) for each group are highlighted in Table A1. The metabolic reconstruction techniques revealed a large number of missing known genes in the Cbl biosynthetic pathways of various environmental bacteria, including those producing B12-dependent methionine synthase (MetH), probably, due to their mutualistic or symbiotic life style [7,12,22,33,65]. Shelton et al. [7] differentiated seven microbial Cba biosynthesis phenotypes based on the presence of complete aerobic biosynthesis (23 genes) or complete anaerobic biosynthesis (25 genes); tetrapyrrole precursor biosynthesis (five genes); combined corrin ring biosynthesis (nine genes); aminopropanol linker (two genes); adenosylation (one gene); nucleotide loop assembly (seven genes); core biosynthesis genes (eight genes). The genomes were grouped into complete biosynthesis, partial biosynthesis (tetrapyrrole precursor and Cbi salvagers), and no biosynthesis [7]. Among 11,000 publicly available bacterial genomes selected from the Integrated Microbial Genomes & Microbiomes (JGI/IMGer) database (https://img.jgi.doe.gov/cgi-bin/mer/main.cgi, accessed on 15 March 2021), only 37% were predicted to have *de novo* Cba synthesis pathways (57% of Actinobacteria and 0.6% of Bacteroidetes) and 58% Cba-producing capacity through the partial biosynthetic pathways, based on the presence of genetic signatures for the lower ligand biosynthesis and attachment [7]. The DMB producers were found in 25% of the 11,000 genomes, and 96 genomes contained one or more *bza* genes, indicating the obligate anaerobes [7]. Twenty four percent of the *bluB*- and *bza*-based evaluated bacteria are predicted to produce the cobamide Cbl required by humans [7]. The species of microorganisms from this database, actively producing Cba, with the identified biosynthesis pathways, are listed in Table A2. Nevertheless, all of them encode Cba-dependent enzymes, indicating the importance of Cba and the precursors salvaging for the bacteria dependent on the nutritional environment [7].

### 3.3. Cobamide Uptake in Eukaryotes

In human and other eukaryotes, including algae, the B12-dependent reactions are the conversion of homocysteine to methionine and interconversion of (2*R*)-methylmalonyl-CoA to succinyl-CoA, which are also provided by the mutualistic microbiota [35,41,66,67]. Approximately 50% of all microalga species obtain Cbl by the way of symbiotic exchange for photosynthate [22]. The differences are that the bioavailable chemical form of B12 to humans has its lower axial ligand as DMB, while some microalgae can use of pseudocobalamin, a Cba with the lower axial ligand adenine, produced by the marine cyanobacteria *Synechococcus* [62]. Among the hundred cyanobacteria genomes screened for the B12 biosynthetic genes, including those involved in the nucleotide loop assembly, pseudocobalamin is the form synthesized by cyanobacteria more broadly. Human gut microbes can also produce the B12 analogues, such as pseudocobalamin of *L. coryniformis* CRL 1001, and/or uptake B12 analogues through structurally distinguished multiple transporters [35,66]. By using the fluorescent Cbl derivatives (fluorophores attached to the ribose position), *Mycobacterium tuberculosis* was found to be able to acquire both cobyric acid and Cbl analogues, whereas a worm *Caenorhabditis elegans* takes up only the complete corrinoid, as well as seedlings of a higher plant *Lepidium sativum* is also able to transport vitamin B12, possibly, by some nonspecific mechanisms [22]. Similarly, the higher fungi, like *Agaricus bisporus,* may transport and accumulate a large amount of B12, which is good alternative diet for vegetarians [21].

## 4. Cobalamin-Dependent Regulation

### 4.1. Cobalamin-Dependent Metabolism

The Cbl-producing strains have Cbl-repressible expression system for co-regulation of siroheme or heme (for sulfite- and nitrite-reducing bacteria), cofactor F_430_ (for methanogenic bacteria), bacteriochlorophyll (for purple α-proteobacteria) and Cbl biosynthesis pathways at the transcriptional level due to their common precursor precorrin-2 and evolutionary complex relationship between tetrapyrrole compounds [7,20,24,68,69]. In addition, the fifteen Cba-dependent enzyme families were predicted in 86% of 11,000 available bacterial genomes [7]. Apart from the traditional view on the function of AdoCbl and MeCbl, such as radical-based rearrangements in mutases, dehydratases, deaminases, ribonucleotide reductases of class II and methyl cation transfers, respectively, a MeCbl-dependent enzyme transferring a methyl anion was determined, as well as AdoCbl was found to be a light sensor (photoreceptor) in the light-dependent regulation of carotenoid biosynthesis [7,58,66,70,71,72]. Moreover, the third class of Cbl-dependent enzymes that use Cbl without an upper ligand were described [70,71,72]. The examples of Cbl-dependent enzymes without an upper ligand may be reductive dehalogenases, epoxyqueuosine (oQ) reductases (QueGs), and the enzymes CblCs responsible for decyanation/dealkylation of Cbl, in which the bound Cbl also lacks a lower ligand [71]. In many methanogenic and acetogenic anaerobes, the B12-dependent methyltransferases as multiprotein enzyme complexes, with one subunit each for binding the B12 cofactor, the methyl donor (methanol, methylamines, and methyltetrahydromethanopterin), and methyl acceptor (coenzyme M and tetrahydrofolate), are involved in growth and energy production. Recently, their repertoire has been extended by the ability to target estrogen as a methyl acceptor, transforming into androgen, with its subsequent degradation during anaerobic bacterial steroid catabolism in a denitrifying bacterium. Bacteria capable of degrading steroids are valuable bioremediation agents due to the industrialization and the ability of some bacteria to synthesis of estrogen (*Gemmata obscuriglobus* and some methanotrophs and myxobacteria), and they are important in the context of host–microbe metabolic interdependencies, including pathogenesis [2]. Within eukaryotes, the vitamin B12 enables to activate a relatively few numbers of enzymes: methionine synthase, and methylmalonyl-CoA-mutase, and class II-like ribonucleotide reductase (RNR) in protists *Euglena* and *Dictyostelium* [73].

### 4.2. Riboswitch-Mediated Regulation

#### 4.2.1. Riboswitch Structure and Mechanism of Regulation

The flux of Cbl itself is regulated by feedback inhibition both by Cbl and corrinoid intermediates, predominantly at the stage of the key enzyme S-adenosyl-l-methionine: UroIII methyltransferase at the substrate UroIII concentrations above 0.2–0.5 µM for such bacteria as *P. denitrificans* and *B. megaterium* [24,26]. However, the Cbl riboswitch-mediated regulation, with the transcription termination and translation control mechanisms of the Cbl-dependent gene expression, are identified in most organisms [24,74,75,76,77].

**Figure 3:**
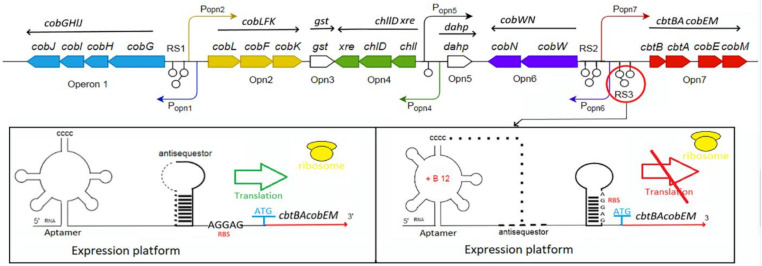
Schematic representation of coenzyme B12 biosynthetic genes cluster I and its riboswitches (RS1, RS2 and RS3) of the *P. denitrifians* ATCC 13867 genome, according to Nguyen-Vo et al. [26]: biosynthetic genes-thick colored arrows; operons-Operon 1, Opn2, Opn3, Opn4, Opn5, Opn6, Opn7; putative promoters-curved arrows; transcripts-straight black arrows above operons. A coenzyme B12-responsive riboswitch (RS3) structure and schematic mechanism of regulation in mRNA is shown below, according to Zhu et al. [76]: CCCC–P3 aptamer domain forming a pseudoknot under a high concentration of coenzyme B12; antisequestor–sequence complementary to RBS and forming a hairpin to attenuate translation; RBS–ribosome-binding site; ATG–strart-codon; *cbtBAcobEM*–sequences of the structural genes of the transcript from Opn7.

A highly structured receptor (aptamer) domain of the Cbl riboswitch binds to the ligand, co-enzyme B12, inducing the secondary and tertiary structure rearrangements along the structure of the downstream regulatory domain (expression platform) containing ribosome-binding site (RBS) (Figure 3).

The complete Cbl-dependent regulatory response lies in the direct control of ribosome loading and indirect modulation of mRNA quantity. At the translation level, the regulatory interdomain kissing loop (L), which is formed in the presence of the bound Cbl (the docking conformation) by the interaction between L5 of the aptamer domain hairpin and L13 of the regulatory domain hairpin, overlaps and sequesters the RBS, thus blocking the translation initiation (Figure 3). Therefore, the frequency of ribosome loading is dependent on the rate of docking between the two loops that form the third kissing loop, which is dictated by the free B12 concentration. At the transcriptional level, the aptamer domain binding with B12 triggers the formation of either the anti-terminator switch or the terminator switch that led to blocking the transcription, thus preventing the coding mRNA synthesis. In the absence of active translation, the abundance of mRNA may be regulated by a combination of cellular factors, either including the *rho*-dependent transcriptional termination or targeting to the transcript the degrading RNAses [74,75,76,77]. However, the riboswitch regulation at the transcriptional level is predominant for the B12-dependent synthesis of the coenzyme B12 [26].

#### 4.2.2. Riboswitch-Encoding Sequences Location and Function

The B12 riboswitch-encoding sequences are located at the 5′-untranslated regions (UTR) of the Cbl operons or/and the leader of Cbl transporters, particularly *btu*B-like sequences [34,66,78,79]. Among available 66 genomes, the two hundred B12 riboswitch-encoding sequences were identified [78]. In silico analysis of the regulatory regions of the coenzyme B12 biosynthetic operons in *P. denitrifians* with the use of Rfam database revealed four riboswitches, three is in cluster I and one is in the cluster II [26]. The first riboswitch (RS1) is common for the Cbl and siroheme biosynthesis and located between the Opn1 and Opn2 operons, which are responsible for the formation of hydrogenobyrinate from precorrin-2 (except for *cobA* and *cobM*) (Figure 1 and Figure 3). Two riboswitches (RS2 and RS3) were found between the operons Opn6 and Opn7, and the riboswitch RS4 was upstream the cluster II [26]. All B12 riboswitches shared the coenzyme B12-binding consensus regulatory and aptamer domains, similar to those of the *btuB* genes in *E. coli* and *S. enterica* [26,80]. Transcription of *cobG*, *cobW*, *cbtB* and *btuB* were shown to be signifiantly suppressed by the coenzyme B12, with the most effect on an auxiliary cobalt transporter CbtBA (43-fold), indicating that the riboswitches should function at the transcriptional level [26,34].

The B12-riboswitch fragments of nearly 200 bp, upstream the genes encoding for an ABC transporter and *cbiB*, were found in the genomes of *P. propionicum* F0230a and *P. freudenreichii* subsp. *shermanii* DSM 20270, respectively. Although it is comprised of a conserved aptamer domain and an expression platform similarly to other riboswitches, the *cbiB* riboswitch from *P. freudenreichii* subsp. *shermanii* DSM 20270 has a definitely different structure, with a short right arm and ‘‘CCCC’’ sequence head responsible for folding of RNA structure, compared to the *btuB* riboswitch of *E. coli* [75,76]. Generally, the reconstruction of B12 regulon and Cbl pathway in most bacterial and archaeal genomes revealed that the most transporters, such as *btu*B and *btu*CDF, are also strongly B12-regulated, if they are not included in the Cbl riboswitch-controlled operon [79]. Upstream of each of five corrinoid biosynthesis operons in *D. restrictus*, the distinct own Cbl riboswitches (Cbl-RS) were found [34]. Its operon-2 was highly up-regulated upon corrinoid starvation both at the transcriptional (346-fold) and proteomic level (46-fold on average), in line with the presence of an upstream Cbl riboswitch [34]. In cyanobacteria, three riboswitch families: namely, cobalamin (Cob): RF00174, adenosylcobalamin (AdoCbl): RF01482 and AdoCbl-variant: RF01689 were upstream of the 462, 338 and 173 genes, respectively, indicating many Cbl-dependent biochemical pathways due to the ability of Cbl to coordinate different upper axial ligands with a diversity of reactivity [71,77]. Although the alternative methionine synthase isozyme MetE is Cbl-independent, the active-transport system for exogenous Cbl uptake, encoded by the *btuB*-*cpdA*-*btuC*-*btuF* operon, was also riboswitch-regulated in a Cbl auxotroph *Synechococcus* sp. PCC 7002, where Cbl was used as the transcriptional attenuator of the *metE* promoter [79]. The expression of *metE*, as well as the genes for S-adenosylhomocysteine hydrolase and serine hydroxymethyltransferase 2, involved in the methionine-folate cycle, were repressed by B12 via a region spanning −574 to −90 bp upstream their start codons in marine and freshwater microalga species [81]. Characterization of the Cbl riboswitch expands the genetic toolbox for the truly autotroph *Synechococcus* sp. PCC 7002 by providing a Cbl-repressible expression system for large-scale industrial applications [77,79].

#### 4.2.3. Ligand Selectivity of Riboswitches

The B12-riboswitches are highly sensitive to the presence of two biological forms, MeCbl and AdoCbl, to regulate expression of proteins involved in the B12 uptake, biosynthesis, or use [72,74]. However, in the absence of such lower ligands as methyl and 5′-deoxyadenosyl groups for formation of MeCbl and AdoCbl, respectively, the free B12 in aqueous solution ligates a water molecule to form hydroxocobalamin (OHCbl) or aquocobalamin (AqCbl) [72]. Both the AdoCbl and AqCbl riboswitches have the similar RNA structure for binding the specific form of B12 at K_D_ of 10–250 nM, with additional peripheral extensions for AdoCbl. The riboswitch regulation by the light-stable AqCbl was suggested to originate in marine bacteria, which were under a high light exposure [72]. Unlike the *E. coli btuB* riboswitch selectively binding AdoCbl, the *cbiB* riboswitch of *P. freudenreichii* subsp. *shermanii* DSM 20270 responsed to various bioactive Cbl besides AdoCbl, such as CNCbl, OHCbl, and MeCbl, and it did not bind pseudocobalamin and light-decomposed vitamin B12 [76]. The plasmid p519-switch-gfp containing 213 bp of the riboswitch sequence, amplified from the leader mRNA of the *P. freudenreichii cbiB*-containing transcript, was adopted as a sensor for the bioactive vitamin B12 quantification in fermented foods, avoiding a sensitivity to nucleic acid and other inactive corrinoids, and pseudovitamin B12 [76]. Recently, the sequence of ligand selectivity determinants related to the key tertiary interaction (J1/3–J6/3 interaction) in the Cbl riboswitches of class II has demonstrated its importance for generating the strong ligand-dependent repression of mRNA expression [74]. Two different classes I and II of Cbl riboswitches share a common four-way junction (P3–P6 helices), forming the core receptor domain, which is responsible for Cbl binding and using various peripheral extensions (P8–P11) to recognize the different Cbl derivatives. The P6 extension is present for the AdoCbl-binding class, but not for MeCbl class [24].

### 4.3. Light-Dependent Regulation

The B12-dependent photoreceptors have been found to sense a light-dependent change in the state of B12 to control expression of genes that were apparently unrelated to B12 metabolism [72]. Many B12-using enzymes, including photoreceptors, bind B12 with a His side chain replacing the DMB ligand, a B12-binding mode known as “base-off/His-on”. The sunlight-induced cleaving of Co-C bond in AdoCbl allows the tetramer of AdoCbl-bound transcriptional regulator CarH to dissociate from DNA, initiating transcriptional activation of the genes responsible for production of light-protective carotenoids. In addition, the corrinoid proteins HgcA and CFeSP bind a “base-off” Cbl and are involved in the methylation of the metals: mercury and nickel, respectively. However, the Co-Cys ligation in HgcA is proposed to facilitate transfer of the methyl group from MeCbl to Hg^2+^ through either transfer of the methyl radical or methyl anion (homolytic or heterolytic Co-C bond cleavage) [71]. After the anionic methyl transfer, Co needs to be re-methylated for the next catalytic cycle by a reductant 2-[4Fe-4S] cluster ferredoxin HgcB. Similarly, AdoMet also can be used as a biological methyl donor to be coupled with Cbl in the enzymatic methylation of a substrate following homolytic Co-C bond cleavage of MeCbl, for example, at the formation of a C-P bond in a bialaphos [71]. In contrast to the B12-induced carotenoid synthesis in many bacteria in response to light, the bacteriochlorophyll-generated singlet oxygen in the purpur bacterium *R. sphaeroides* triggers an expression of the protective genes responsible for the photooxidative stress. The B12-dependent synthesis of bacteriochlorophyll (and pigment-binding proteins) occurs when enough tetrapyrroles for the synthesis of B12, suggesting that a homologue of a photosynthesis repressor AerR, a B12-binding antirepressor of sensor CrtJ to light and redox control, may bind B12 rather than heme in the methylation step, from protoporphyrinogen IX to Mg protoporphyrin monomethylester.

## 5. Evolution and Role of B12 Auxotrophy

### 5.1. Marine Microbial Community Auxotrophy

In Nature, the vitamin auxotrophy constitutes frequent bacterial phenotype in aquatic, terrestrial and gut ecosystems, that together with amino acid auxotrophy evolving reduction of the metabolic burden stemming from the production of energetically costly compounds [33]. This leads to the mutualistic life style and mosaic metabolic interdependencies among bacteria and other organisms to ensure access to such essential biomolecules [82]. The reduction of genes in free-living organisms, particularly the open-ocean bacterioplankton, appears to be driven by natural selection rather than drift, but makes them dependent on co-occurring microbes for lost metabolic functions [83]. The B12 auxotrophy in microalgae was shown to originate multiple times through independent evolution in the phylogenetically unrelated species. The suggested mechanism was the B12-independent MetE gene loss or its transformation into the pseudogene due to the continuous repression of the *metE* transcription in the presence of Cbl in the environment [84]. Metabolomes of the pseudocobalamin-producing N2-fixing marine cyanobacterium *Trichodesmium* and its microbiome are highly synchronized by cycling of carbon, nitrogen, iron, and Cbl. The transcriptional patterns, related to the Cbl pathways orthologous groups, revealed that the DMB production by *Trichodesmium* controls the subsequent use of Cbl by microbiomes that drives the Cbl auxotrophy and community structure of both the host holobiont’s colonies and microbiome [16]. This role would distinguish *Trichodesmium* from other cyanobacteria that solely produce pseudo-B12, which certain algae can remodel to a bioavailable form, implying a complex B12 cycle between cyanobacteria and microalgae in the photic ocean zone [62]. The DMB biosynthesis gene, encoding the Cbl-requiring enzyme BluB in *Trichodesmium*, may have been acquired horizontally much later as evidenced by its clustering within Proteobacteria [17]. However, it was suggested that the first cyanobacterium cell, which synthesized chlorophyll, was a Cbl-dependent heme-synthesizing diazotrophic anaerobe, considering the source genes from which several steps in chlorophyll biosynthesis are derived and the cofactor demands of the pathway [85]. In addition, the iron metabolism genes *cbi*X (Tery_4741) and *isi*B (Tery_1666) were found within the Cbl biosynthetic pathway, suggesting a potentially unrecognized role for iron limitation in simultaneously affecting *Trichodesmium* photosynthesis, nitrogen fixation, and B12 biosynthesis [17]. The future oceans are predicted to be warmer, higher in CO_2_, and to have expanded oligotrophic regions with the active participation of the *Trichodesmium*-dependent consortium that would considerably affect marine biogeochemical cycles [16,17].

### 5.2. Gut Microbial Community Auxothrophy

**Figure 4:**
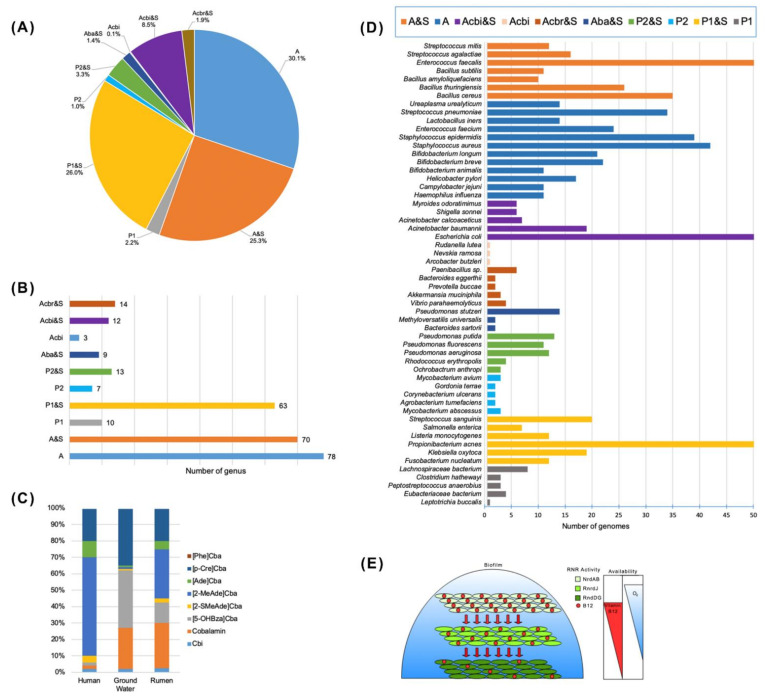
Distribution of microbial cobalamins (vitamin B12 family cofactors) biosynthesis capability in various ecosystems: (**A**) general distribution of the basic B12-related pathway variants among publicly available human gut microbial (HGM) genomes, according to Rodionov et al. [12]: **P1** and **P2**-*de novo* anaerobic and aerobic Cbl biosynthesis (protothrophy), respectively; **P1&S** and **P2&S**-*de novo* anaerobic and aerobic biosynthesis and salvage; **Aba&S**-cobyrinate auxotrophy and salvage; **Acbr&S**-cobyrinate diamide auxotrophy and salvage; **Acbi&S**-cobinamide auxotrophy and salvage; **A&S**-cobalamin/B12 auxotrophy and salvage; (**B**) distribution of the basic B12-related pathway variants at the microbial genera level (number of genus); (**D**) distribution of the basic B12-related pathway variants at the species level (number of genomes from five and more strains of a species); (**C**) distribution of cobamides in human, ground water and rumen, according to Degnan et al. [66]; (**E**) Distribution of vitamin B12 availability and ribonucleotide reductase activity in the *P. aeruginosa* biofilm-forming cells according to the experimental data of Crespo et al. [58]: vitamin B12 is indicated by red color; oxygen concentration gradient is in blue; ribonucleotide reductase (RNR) activity is indicated by green color: oxygen-dependent class Ia RNR (NrdAB) and oxygen-sensitive III RNA (RnrdJ) are B12-independent, while oxygen-independent class II RNR (NrdJ) is highly B12-dependent and important for providing the cells with deoxyribonucleotide triphosphates (dNTP) during the biofilm growth.

A lot of gut microbiota (≥20% by abundance) have auxotrophic phenotype for the B group vitamins, with the highest level of vitamin B12 auxotrophy (50–80% auxotrophs and multi-auxotrophs) that implies their strong dependence on the exogenous supply of this micronutrient from the diet and/or via syntrophic sharing between prototrophic and auxotrophic species [12,66,86]. Generally, the B vitamins sharing promotes stability of normal gut microbial community, with in vivo predominance (by relative abundance) of such genera as *Bacteroides* (∼50–60%), *Flavonifractor* (∼30–40%), *Escherichia* (∼20%), *Akkermansia* (≤20%), *Peptoniphilus* (≤20%), *Clostridium* (∼15%), *Pseudoflavonifractor* (≤15%), *Finegoldia* (≤15%), *Phascolactobacterium* (≤15%), *Parabacteroides* (≤15%), *Agathobaculum* (≤15%), *Egerthella* (≤10%), *Dorea* (≤10%), *Neglecta* (≤10%), *Blautia* (≤10%), *Enterococcus* (≤10%), *Hungatella* (≤10%), *Lachnoclostridium* (≤10%), *Catabacter* (≤10%), *Anaerotignum* (≤10%) [86]. The B12-producing bacteria (protothrophs) were found among *A. muciniphila*, in which only one out from four reference genomes has *de novo* B12 synthesis pathway while the other three strains rely on the B12 salvage (auxotrophy) [86]. Only three out of ten *L. reuteri* strains had the B12-producing phenotype some of which could synthesize B2. A single vitamin B12-producing phenotype (auxothrophs by other B-vitamins) was found in *B. xylanisolvents*, *K. oxitoca*, *P. studzeri*, *S. parasanquinis* [12]. In general, vitamin B12 is mainly synthesized by the species from three orders Propionibacterales, Corynebacterales, Coriobacteriales belonging to Actinobacteria, and the orders Clostridiales, Selenomonadales and Veillonellales from Firmicutes [7,12,86]. The capability of B12 biosynthesis was also predicted earlier in nearly 40% of the human gut microbial genomes: all Fusobacteria, and rarely Actinobacteria, Proteobacteria, Bacteroidetes, Firmicutes [13]. Meanwile, more than 80% of the B12 auxotrophs (1235 among 2228 strains) lack all the B12 biosynthetic enzymes, except for BtuR/PduO family adenosyltransferases converting the exogenous Cbl into the bioactive AdoCbl (Figure 4A,B,D; Table A1). B12 was not essential for growth of 17 auxotrophic species, including 9 *Staphylococcus* spp., without the B12-dependent enzymes, and *B. cereus* that has both the B12-dependent MetH and B12-independent analogue MetE [12]. Six B12-requiring strains of Bacteroidales, such as *Collinsella aerofaciens* and *Clostridium scindens*, possessing a complete set of B12 synthesis genes were rather the precorrin-2 auxotrophs than B12-dependent. However, the experimentally described and bioinformatically predicted vitamin B12 requirements for the growth have been shown for the human gut bacteria, namely: *R. bromi, C. spiroforme, S. marcescens, S. fonticola, Sh. sonnei, Sh. flexneri, Sh. dysenteriae, E. fergusonii, E. coli, L. sakei, L. delbrueckii, B. thetaiotaomicron, B. ovatus, B. caccae* [12]. Remarkably, the provision of excess B-vitamins (∼30-fold above normal) did not influence on the frequency of auxotrophs in the gut microbiota in vitro (fecal samples) or in vivo [86]. Degnan et al. [66] showed the similar distribution of Cbl and Cbi, and other corrinoids inherent for microbial communities in dependent on their habitats, from human to rumenants and ground water (Figure 4C). The gut microbes have been suggested to be under reductive genomic evolution, driven by genetic drift, which is common in endosymbiotic bacteria and states that fitness gain accompanying gene loss is frequency dependent, demanding the B-vitamin donors must remain in sufficient abundance in communities to ensure that auxotrophs are not subject to negative selection [83].

As for the gut microbiota of ruminants, the majour species *Selenomonas ruminantium*, *Megasphaera elsdenii*, *Butyrivibrio fibrisolvens, Prevotella* spp. and some unidentified species, which habitat the rumen, have been shown to provide the largest amounts of the coenzyme B12 and analogues, while the increase in *Bacteroidetes*, *Ruminiclostridium*, *Butyrivibrio*, and *Succinimonas* and Succinivibrionaceae species correlate with lower concentrations of vitamin B12, indicating their complete or partial auxothrophy [87]. Ruminants have higher vitamin B12 requirements than nonruminants due to their active propionic acid metabolism; therefore, the arising B12 requirenment is satisfied by an increase in Co^2+^ supplementation for stimulation of B12 biosynthesis in their mutualistic bacteria [87].

### 5.3. Evolutionary Strategy for Cosmopolite Bacteria Auxothrophy

A widespread organotrophic anaerobe Thermotogales, a habitant of mainly high-temperature hydrocarbon-enriched ecosystems, can synthesize vitamin B12 de novo from glutamate [44]. This capability has been suggested to be acquired with the requisite genes from distantly related lineages. Thus, the *Thermosipho* species have two gene clusters: the corrinoid synthesis and Cba salvage genes acquired horizontally from the ancestral Firmicutes and consortium of bacteria or Archaea, respectively [44].

However, some auxotrophy can be observed for cosmopolite bacteria to reduce energy cost by their different cells’ populations under different stress factors, despite the complete de novo B12 synthesis signatures in the genomes.

This was found within the populations of *P. aeruginosa* PAO1 cells during their division through dNTP synthesis in the planktonic (aerobic) and biofilm (anaerobic) conditions [58]. The *P. aeruginosa* ribonucleotide reductase NrdJ (class II RNR) is simultaneously oxygen-independent and vitamin B12-dependent, while B12 is only aerobically synthetized in *P. aeruginosa*. The class II RNR activity was observed in both conditions in the presence of vitamin B12 independently of B12-riboswitch regulation, contrarily to other vitamin B12-dependent enzymes belonging to the methionine and Cbl biosynthesis and some RNR from other microorganisms. Crespo et al. [58] suggested that the external cells in the *P. aeruginosa* biofilm continue to contact with aerobic environments that allows producing and delivering Cbl into the deeper layer of the biofilm-associated cells (Figure 4E). Cbl diffuses through the biofilm, creating a coenzyme B12 concentration gradient along its structure, and activates the class II RNR in the microaerophilic conditions, where the class Ia or class III RNRs are inactive [58].

### 5.4. Mammalian B12 Auxotrophy

The essential metabolites of the B12 coenzyme family for mammals are synthesized only by gut microbiota [66]. However, the microbial production of vitamin B12 plays only a limited role because of its restricted availability in the environment, including ruminants particularly juveniles [87]. This can only result from lysis of the microbes that produce B12 or specific behaviors of the hosts, such as coprophagy [6]. The human B12 auxotrophy evolutionary compensated by eating lean red meat or developing the usage of fermented food, where lactic acid bacteria provide a variety of essential intercellular vitamins by sporulation, which immediately liberates the cytoplasmic content of the parent cells. A consequence for biotechnology applications is that, if valuable for their host, B12-producing microorganisms should be sensitive to bacteriophages and colicins, or make spores to be addicted to lysis [6]. Nevertheless, the human B12 metabolic pathway contributes to the susceptibility to vitamin B12 deficiency that has genetic associations with diverse ethnic populations or vitamin B12-related chronic diseases. It has been identified the significant associations of vitamin B12 status with 59 B12-related single nucleotide polymorphisms (SNPs) from 19 genes, including co-factors or regulators for the transport of vitamin B12 (*FUT2*, *FUT6*, *MMACHC*, *TCN1* and *TCN2),* membrane transporters actively facilitating the membrane crossing of vitamin B12 (*ABCD4*, *CUBN* and *CD320),* the catalysts of enzymatic reactions in the one-carbon cycle (*CBS, MTHFR* and *MTRR),* a cell cycle regulation (MS4A3), mitochondrial proteins (*CLYBL*, *MMAA* and *MUT*) and the genes of unknown function (*ACTL9*, *CPS1*, *DNMT2*/*TRDMT1* and *PON1)* [1,88].

## 6. Cell Factories for Cobalamin Production

### 6.1. Discovery and Development of Biotechnological B12 Producers

The discovery in 1948 by Rickes and colleagues of a Cbl synthesized in *Streptomyces griseus* led to employment of several fermentation processes and their modifications on an industrial scale [89]. A *Streptomyces olivaceus* strain that was used primary for antibiotic production showed to produce vitamin B12 as a byproduct [90]. The detailed studies on vitamin B12 production with *S. olivaceus* were found it to be applicable for the enrichment of animal feeds. Then, the initial yields of vitamin B12 (up to 3 µg/mL) produced by this microorganism were increased by influence of Co^2+^ and various nitrogenous nutrients, such as distiller’s solubles of wheat/corn/milo, soybean meal, and penicillium mycelium [90,91]. Over the years, a variety of microorganisms, mainly the strains of aerobic *P. denitrificans, S. meliloti, B. megaterium* and anaerobic *S. typhimurium, Propionobacterium* spp. were reported to produce significant titers of B12 in different fermented media [9,28]. Detailed studies on vitamin B12 pathways resulting in its availability intracellularly or extracellularly on an industrial scale have been carried out using the batch or fed-batch process by *Propionibacterium* sp. and *P. denitrificans* under anaerobic and aerobic conditions, respectively [6,54]. The higher yields of Cbl were gradually achieved through optimization of the culture medium and fermentation process (addition of Co^2+^, DMB, ALA, amino acids, and vitamins), mutagenesis of the producing strains (UV light, ethyleneimine, nitrosomethyluretane or *N*-methyl-*N*′-nitro-*N*-nitrosoguanidine), overexpression of the gene clusters, which involved in or interrelated with Cbl biosynthesis, optimization of their promoter and/or riboswitch-mediated regulation, ribosome engineering technology, and B12-auxotrophy evolution (Table 2). The vitamin B12 yields up to 15 μg/mL were reported for the natural strains, whereas genetic engineering and selection of the highly producing mutants could increase the vitamin B12 yields up to 300 μg/mL at industrial scale [54,92].

#### 6.1.1. Propionobacteria

The *Propionibacterium* species are widely used in production of dairy products (cheese) and industrial production of propionic acid, lactic acid and vitamin B12 [25]. The major advantage of *Propionibacterium* spp. is capability of the use various industrial waste products as substrates to synthesize the metabolites, and their strains have been admitted as GRAS (generally recognized as safe) by the U.S. Food and Drug Administration since they are not known to produce endotoxins [54]. After isolation and detailed characterization of the genes involved in the vitamin B12 biosynthesis in *P. freudenreichii* and *P. shermanii* strains, they became possible to increase the Cbl production. Kiatpapan and Murooka [93] and Piao et al. [94] reported accumulation of precursors (ALA and tetrapyrrole compounds) in the Cbl biosynthetic pathway of *P. freudenreichii* via series of constructed expression vectors containing the *cob* and *cbi* gene families. The recombinant *P. freudenreichii* IFO12426 strain resulted in an increase of vitamin B12 up to 1.46 mg/L [94]. When Piao and colleagues [94] added the *hem* genes from *R. sphaeroides,* the yield of B12 became to be 1.68 mg/L. The studies on media optimization for *Propionibacterium* species showed that selected carbon sources, addition of amino acids, minerals, precursors, vitamins and vitamers (chemical vitamin analogues), blue light, co-fermentation and co-cultivation affect Cbl production [95,96,97,98,99] (Table 2). Thus, the concentration of 58.8 mg/L and production of 0.37 mg/g of vitamin B12 were obtained with an expanded bed adsorption bioreactor by using the propionic acid and DMB control method [97]. Co-fermentation of glycerol and glucose with a gradual addition strategy for anaerobic co-production of propionic acid and vitamin B12 by *P. freudenreichii* gave the yields 0.71 g/g of propionic acid and 0.72 mg/g of vitamin B12, and the productivities 0.36 g/L h of propionic acid and 0.36 mg/L/h of vitamin B12, respectively [97]. Blue light and some microbial B12 analogues increased twofold productivity of B12, possibly, due to removing the B12-dependent riboswitch inhibition in the emerged resistant *P. freudenreichii* clones [98,99].

Co-cultivation of *P. freudenreichii* with *L. brevis* on a pH-regulated wheat bran can be a promising way to produce vitamin B12 fortified plant food ingredients that simultaneously inhibits the growth of total Enterobacteriaceae [100] (Table 2). Chamlagain et al. [96] studied the B12 production capacity of *P. freudenreichii* and *P. acidipropionici* strains in a whey-based medium, with supplementation of riboflavin and nicotinamide. Riboflavin and niacin are known to be precursor for DMB and responsible for DMB transformation, respectively. Using these knowledges, the maximum of vitamin B12 in the *P. freudenreichii* and *P. acidipropionici* strains was reached up to 712 ng/g (4-fold) in comparison to the control cultures [96].

**Table 2:** Selected strains for vitamin B12 fermentation process.

Strain/Pathway	Strategy/Tactic	Main Precursors	Product Yield	Reference
*P. denitrificans* SC510, aerobic/salvage	Random mutagenesis using radiation (UV light) and chemicals (ethyleneimine and nitrosomethyluretane), overexpression of *cobF-cobM* gene cluster as well as *cobA* and *cobE* genes; optimization of promoters, RBSs, terminators.	sucrose, betaine, DMB	214 mg/L	[101]
*S. meliloti* MC5-2, aerobic/salvage	Random mutagenesis based on atmospheric and room-temperature plasma (ARTP); overexpression of *hemE*; deletion of *cobI*, and usage of a riboswitch based on *butB* element from Salmonella typhimurium in front of a *gfp* reporter gene driven by the constitutive promoter *PmelA*	sucrose, DMB	156 mg/L	[23]
*Pseudomonas* sp. PCSIR-B-99, aerobic	Optimization of fermentation process	methanol, DMB	3500 μg/L	[89]
*S. olivaceus* NRRL B-1125, aerobic	Optimization of fermentation process	glucose, DMB	1–3.3 µg/mL	[90]
*P. shermanii*, anaerobic	Overexpression of biosynthetic genes	glucose, DMB	206 mg/L	[54]
*P. freudenreichii* CICC 10019	Optimization of fermentation process	glucose, corn extract, DMB	58.8 mg/L	[95]
*B. megaterium*, anaerobic	Overexpression of *hemACDBL*, *sirA*, *cbiXJCDETLFGA*, *cysGA*, *cbiY*, *btuR*, *glmS*, *metH*, *rtpR* with xylose-inducible promoter; antisense RNA for *hemE*, *hemZ*, *sirB*. Bypassing of the B12 riboswitch	glucose; ALA; DMB	0.220 mg/L	[102]
*B. megaterium*, wild strain, anaerobic	Optimization of fermentation process	glucose; ALA; DMB	204.46 µg/L	[103]
*E. coli*, salvage	The 22 native *cob* genes located in six operons from *P. denitrificans* ATCC 13867 were PCR-amplified and cloned in three compatible plasmids under the strong inducible T7 promoter	ALA	0.65 µg/g	[104]
*E. coli*, aerobic/anaerobic/salvage	Optimization of fermentation process; expression was conducted by assembling six modules comprising 28 genes from *R. capsulatus*, *B. melitensis*, *S. meliloti*, *S. typhimurium*, and *R. palustris*	ALA; glycine, succinic acid, betaine	307 µg/g	[105]
*P. freudenreichii* IFO12426	Optimization of fermentation process, overexpression of hem genes from *R. sphaeroides*	glucose; ALA	1.46 mg/L	[94]
Mesophilic methane bacteria from digested sludge	Optimization of fermentation process: enriching trace metal salts by an electrolysis process	H_2_/CO_2_ medium (biogas or coal gas)	185 mg/L	[106]
*S. meliloti* CGMCC 9638 aerobic/salvage	Optimization of fermentation process (9–12 days)	sucrose, glycine betaine, corn liquor, DMB	at least 50 mg/L (up to 180 mg/L)	[107]
*P. freudenreichii* (food- grade)	Aqueous cereal-based matrices fermentation	malted barley flour, riboflavin, nicotinamide, cobalt	712 μg/kg	[96]
*P. freudenreichii*	Feed-back inhibition of propionic acid	glucose, DMB, corn steep liquor, cobalt	59.5 mg/L (0.59 mg/L/h)	[97]
*P. freudenreichii* DSM 20271	Co-fermentation with *L. brevis* ATCC 14869 with pH control (pH 5.0)	wheat bran dough and water (15:85)	332 ± 44 ng/g dry weigth (3 days)	[100]

Cobalt ions are also important factor for the optimum growth and vitamin B12 production (Table 2). The Co^2+^ addition (5 μg/mL) in a whey-contained medium could increase the level of vitamin B12 three-fold in the *P. freudenreichii* strain DF13. Interestingly, that higher than 5 μg/mL Co^2+^ levels did not increase the yield in the study by Hugenschmidt et al. [92]. The improvement of Cbl biosynthesis in *Propionibacterium* was also performed via the usage of ribosome engineering technology, which aimed at activation of silent genes involved in a bacterial metabolite production. Tanaka et al. [108] reported introduction of mutations into the antibiotic resistance genes Rif^R^, Gen^R^, and Ery^R^ in *P. shermanii,* which were shown to be effective at increasing the levels of expression of genes involved in the enhancement of vitamin B12 yield fivefold, although net production (μg/L) was unchanged due to reducing the mutant cells.

#### 6.1.2. Pseudomonades

*Pseudomonas* spp. are known to be capable of the industrial production of various organic compounds, including vitamin B12 (Table 2). They occur in various ecosystems, mostly using oxygen as an electron acceptor for their metabolic reactions [54]. Among *Pseudomonas* spp., the strains of *P. aeruginosa* and *P. putida* were described as the better Cbl producers, but the pathogenicity of these strains complicates their usage in pharmaceutical and food industry for human needs [54,109]. Attempts to study the Cbl biosynthesis were conducted by the Merck company on strain *P. denitrificans* MB580 and, later, through several random mutagenesis steps by Cameron and colleagues [110], resulted in a high-producing Cbl strain SC510. Later, once the researchers from Rhone-Poulenc-Rorer (RPR) could disclose the elucidation of the complete biosynthetic pathway of vitamin B12 in *P. denitrificans*, using set of metabolic engineering and mutations (Table 2), they successfully obtained *P. denitrificans* strain producing above 200 mg/L of vitamin B12 [25,105]. Recently, the whole lengths of riboswitches in the Cbl cluster I were completely removed, and the promoters regulated by riboswitches were replaced with strong constitutive promoters that led to the improved 2-fold B12 biosynthesis in the strain *P. denitrificans* ATCC 13867 [26]. Since *Pseudomonas* spp. are able to utilize different carbon and nitrogen sources from the environment, the attempts to improve of the vitamin B12 production were conducted on their different species under limited conditions [54]. A strain *Pseudomonas* sp. PCSIR-B-99, with profound ability of methanol utilization as carbon and energy source, was found to be an excellent producer of vitamin B12 on a modified basal medium (Table 2). The works of Riaz et al. [89] investigated the parameters affecting the vitamin B12 production in *Pseudomonas* sp. PCSIR-B-99, such as time of fermentation, different concentrations of methanol and cobalt ions effect on the growth. The maximum of vitamin B12 production of up to 3500 μg/L was observed in the medium containing 3.5% (*v/v*) methanol, 1.0 mg/L of Co^2+^, and 200 mg/L of DMB after 72 h of fermentation [89]. However, the Cbl biosynthesis in these strains is complicated due to the long fermentation process (up to 180 h), complex and frequently expensive media composition, and lack of suitable genetic techniques for the strain engineering that required searching the novel bacterial hosts for the Cbl-overproducing-based genetically engineered constructions.

In recent years, many studies were performed and observed by various scientific groups on the vitamin B12 biosynthesis in the members of *Bacillus* spp., *E. coli, Rhizobium* spp., *Lactobacillus* spp. and others [24,54]. Since the genes required for Cbl biosynthesis and their regulative elements were able to be replaced or inserted into replicating plasmids, which are responsible for the synthesis of targeted proteins, they have been applied to the different prospective host organisms for their heterologous expression in the nature producer *B. megaterium,* as well in *E. coli*, which is non-producing *de novo* B12 in nature [105,108].

#### 6.1.3. Bacillus megaterium

In the last decades, *B. megaterium* and *E. coli* have been well-studied platforms for the industrial production of various important metabolites due to their ability to secrete proteins directly into the growth medium for 24–48 h [10,52,105]. In contrast to the *E. coli*-like bacteria, *B. megaterium* does not produce endotoxins associated with the outer membrane, which combined with its growth on a variety of carbon sources and simple media has made its suitable for food and pharmaceutical production processes [52]. The strain *B. megaterium* DSM319 was shown to be able to grow on inexpensive carbon sources, such as raw glycerol from biodiesel production that makes this organism an ideal production host. In addition, the cobalt bioavailability was allowing to increase the B12 yields from 2 to 13 µg/L for the parent strain, and bypassing of the nature B12 synthesis regulation system by cloning led to even higher yields, ≥220 µg/L [54,102]. Mohammed and coauthors [103] reported increasing B12 production up to 759-fold using optimum conditions for the fermentation process of *B. megaterium* based on statistical design (Table 2). Downregulation or elimination of the concurrent biosynthetic pathways, such as heme and siroheme production, are the common genetic strategies for the redirection of their precursors toward the vitamin B12 biosynthesis. Thus, induction of the heme synthetic branch at silencing of *hemZ* encoding for coproporphyrinogen III oxidase in *B. megaterium* DSM509 led to the 20% increase (from 0.26 in the wild-type to 8.51 µg/L in engineered strains, respectively) of the vitamin B12 concentration intracellularly in the works of Biendieck et al. [10]. Fang and colleagues via inhibition of the genes, *hemE* and *hemH,* could improve the precorrin-2 intermediate production using sRNA-mediated approach [24].

#### 6.1.4. Escherichia coli

The salvager *E. coli* that has only the genes responsible for the exogenous B12 precursor uptake is another promising host for the vitamin B12 production due to its growth for 24 h, instead of 180 h for the complete fermentation in *P. denitrificans*, and capability of synthesizing ALA that plays key role as a precursor in the tetrapyrrolidine formation [24,104]. Ko et al. [104] reported the successful Cbl biosynthesis in *E. coli* harboring three compatible plasmids with the cloned 22 Cbl genes from six operons of *P. denitrificans*. These modifications led to 0.65 µg/g of vitamin B12 (Table 2). Moreover, the B12 synthesis in *P. denitrificans* is strictly aerobic, but the recombinant *E. coli* can produce B12 under both aerobic and anaerobic conditions. This suggests that the oxygen-dependent B12 biosynthesis in *P. denitrificans* might be due to some regulatory mechanisms in those native hosts [24,101]. Later, a total of 28 genes from *R. capsulatus, B. melitensis, S. meliloti, S. typhimurium*, and *Rhodopseudomonas palustris* that are divided into six engineered modules were expressed in *E. coli* strains to synthesize *de novo* vitamin B12 and to assemble the nucleotide loop simultaneously by the recombinant CobU, CobT, CobS, and CobC and via the salvage pathway, achieved by the endogenous *E. coli* adenosyltransferase BtuR [105]. The additional knockdown of the heme biosynthetic gene expression, combined with optimal fermentation conditions, could increase the vitamin B12 productivity of the recombinant *E. coli* strains from 1–2 to 307.00 µg g^−1^, which is still much less than the industrial strains of *P. denitrificans* (214.3 mg L^−1^) and *P. freudenreichii* (206.0 mg L^−1^), but is comparable with the wild-type strain of *B. megaterium* and its engineered strain (0.26 and 8.51 µg L^−1^, respectively), and the wild-type *P. denitrificans* (2.75 µg g^−1^) [105]. Recently, an auxotrophic selection strategy for improved production of coenzyme B12 was successfully developed for *E. coli* [64]. Given that the expression of coenzyme B12-independent methionine synthase gene *metE* is repressed by the presence of vitamin B12, the prolonged repression by a reliable source of the vitamin could lead to accumulation of mutations and eventually gene loss [81]. To select a highly effective producer, the gene *metE* was deleted in *E. coli*, thus limiting its methionine synthesis to only that via coenzyme B12-dependent synthase encoded by *metH* [64].

#### 6.1.5. Other Microorganisms

Some microorganisms are also known as producing B12 vitamin but are less attractive for the industry due to their complex and specific requirements to cultivation conditions, such restrictions as low growth rates, presence of oxygen or utilization of limited carbon sources. Among them, the vitamin B12 family cofactors can be synthesized by the soil, marine microorganisms, and microflora from the digestive tracts of humans and animals [25]. *Rhizobium* spp. isolated from a forest soil demonstrated a potential function to produce Cbl. Possible roles of Cbl in interactions of plants with the bacteria are still remain unknown or little known, though Cbl is not required for the plant metabolism [111]. However, some cases of the vitamin B12 utilization appeared to exist in the seedlings of *Lepidium sativum*, possibly, transporting Cbl for their microbial endosymbionts [22]. Mutation of the soil *S. meliloti* MC5-2 was optimized using helium-based atmospheric and room temperature plasma technique (ARTP) and reaching the yield of 156 mg/L vitamin B12 that is 22% higher than that of the wild-type strain (Table 2). Moreover, it was found that the nature genetic B12 regulatory element responsible for the Cbl derivatives uptake (the expression of transport protein BtuB), is sensitive for increasing the amount of intracellular vitamin B12 [23]. Among lactic acid bacteria (LAB) that excretes lactic acid as the major end product and generally recognized as GRAS organisms, *L. reuteri* was shown to produce pseudovitamin B12 that is inactive for humans but important for their friendly microbiota [25,51,54]. Biosynthesis of Cbl in the marine environment has also been observed and reported. The marine microbes producing Cbl are likely the predominant source for microbial assimilation in the Ocean surface and association with phytoplankton blooms [17]. The major producers of the vitamin B12 family cofactors in the Ocean are heterotrophic Protobacteria, chemoautotrophic Thaumarchaeota and Cyanobacteria, which supply fixed carbon and nitrogen affecting primary marine and biogeochemical cycling [14]. Proteobacteria and Thaumarchaeota produce Cbl, while majority of Cyanobacteria, such as *Synechococcus, Spirulina, Prochlorococcus*, *Aphanizomenon* and others *(>95%)*, that are the most abundant phytoplankton on Earth, supply and use pseudocobalamin, due to the absence of the BluB protein that synthesizes the α-ligand DMB [17]. The bluB-containing Thaumarchaeota, however, solely produce Cbl, demonstrating that DMB activation may occur through a similar enzyme, with a high sequence divergence or through a different, yet unknown, genetic mechanism [14].

## 7. Industrial Bioprocess of Vitamin B12

Vitamin B12 is currently produced industrially by microbial fermentation, using genetically engineered strains of *P. denitrificans* and *P. freudenreichii* (Table 1). The chemical production of B12 is a complex and economically unviable process, involving 70 reactions, whereas biotechnological synthesis occurs via few enzymatic steps, followed by conversion of the natural vitamin B12 into the air-stable CNCbl form through a reaction with cyanide (during industrial manufacture) [5,54]. For human and animals, vitamin B12 is used as a part of nutrition in various forms of food/feed supplements and grade. According to the Dietary Reference Intakes (DRIs) developed by the Food and Nutrition Board (FNB, 1998), the average of recommended daily allowance of vitamin B12 is about 2.5 µg for healthy person [54]. As feed additives, vitamin B12 is mainly used for pigs, poultry and calves, suggesting that their own microbiota does not provide sufficient amount of the coenzyme in contrast to ruminants [6,54]. However, ruminants also mostly need to uptake the exogenous vitamin B12 [66,87]. The dosage levels of all animal feeds in Europe and US lies between 10 and 30 mg/t. Today manufacturing of the biotechnological vitamin B12 is more than 30 t/year for food, feed and pharmaceutical applications [54]. The dominant producers of vitamin B12 on the market are the Chinese CSPC Huarong Pharmaceutical Company, North China Pharmaceutical Company, Hebei Yuxing Bio-Engineering Company, Ningxia Kingvit Pharmaceutical Company, Henan Lvyuan Pharmaceutical Company, and Sanofi-Aventis Company (France). Industrial bioprocesses of vitamin B12 with *P. freudenreichii* have successfully been established through rational and classical metabolic engineering with the maximal reported yields of 206 mg/L [54,105]. Moreover, *Propionobacterium* species are widespread in production of fermented products. Recent year’s usage in situ fermentation by the safe GRAS *P. freudenreichii* allows making enrichment of the food products with an active vitamin B12 [27,54,92]. Chamlagain and coathors [96] demonstrated the potential of in situ production of active B12 in food matrices using the strains of food-grade *P. freudenreichii* to enrich foods with B12 naturally. Such products have advantages since the vitamin B12 is already bioactive for human and does not require the extraction and purification steps, as it is necessary for industrial biosynthesis [92].

The production of vitamin B12 at an industrial scale with *P. freudenreichii* strains is performed under anaerobic conditions but in the presence of oxygen for DMB ligand synthesis [25,54,106]. Thus, the vitamin B12 bioprocess using *Propionibacterium* strains usually is divided into two steps: (1) bacterial cells cultured under anaerobic conditions for the first three days to generate AdoCbi (intermediate lacking DMB ligand), and (2) synthesis of lower ligand followed by conjugation to cobinamide under delicate airing of the culture for up to three days. Generally, the optimum temperature of the process sets at 30 °C [25,54,106]. The active Cbl produced by *Propionobacterium* strains are accumulated mostly during stationary phase. As carbon sources for industrial fermentation *Propionobacterium* strains can use lactate, glucose, sucrose, fructose (from sugar beet molasses) at concentrations of 50–100 g/L [112] but cannot utilize maltose [113]. Other carbon sources, which *P. freudenreichii* can metabolize, are mannose, glycerol, inositol and galactose [112,114]. However, two subspecies of *P. freudenreichii* should be distinguished by capability of lactose fermentation for *P. freudenreichii* subsp. *shermanii* and nitrate reductase activity for *P. freudenreichii* subsp. *freudenreichii* [114]. On the other hand, *P. acidipropionici* metabolizes diverse carbon sources, including sucrose and maltose [55]. The supplemented compounds to enhance culture growth can include such nutrients as yeast extract, corn steep liquor, casein in the concentration 50–70 g/L of hydrolysate. The components stimulating the fermentation process comprise the small concentrations of minerals (iron, magnesium, manganese, cobalt salts), vitamins (betaine, choline, riboflavin, nicotinamide), amino acids (glutamic acid, glycine, threonine), ALA, and the B12 precursor DMB of 10–25 mg/L [54]. The key supplements Co^2+^ and DMB significantly affect an industrial B12 production [97,101]. For the industrial B12 production, DMB is supplemented after the first 3 days of anaerobic fermentation and then shifted to an aerobic incubation for another 3–4 days [9]. The earlier addition of DMB has been shown to suppress the growth and B12 production. However, DMB cannot be added to foods [96]. The important step of bioprocess is control of propionic and acetic acid amounts by its subsequent removal. An excess of propionate is inhibitory for the growth of *P. freudenreichii* and B12 production and that is why the accumulated acids are usually neutralized in commercial B12 manufacturing [9,96]. Therefore, necessary for the production to maintain a culture at a range of pH 6.5–7 is to neutralize the accumulated propionic and acetic acid, when they reach to 10% of the fermentation volume [9,25]. The relative proportion of the acids is also affected by the carbon substrates and the presence of oxygen. To reduce the inhibitory concentrations of the rapid accumulation of acids, particularly due to the vectors enhancing propionic acid and ALA precursor, the fermented culture can be changed to aerobic and, after, back to the anaerobic conditions allowing increasing the Cbl yield from six to 12 mg/L [54,115]. Alkalization of the media, “cross-flow” filtration, extraction fermentation and others are also been reported to remove propionic and acetic acids. However, propionate and acetate are beneficial as antifungal agents to improve the storage period of the products in food fermentation [25,54].

**Figure 5:**
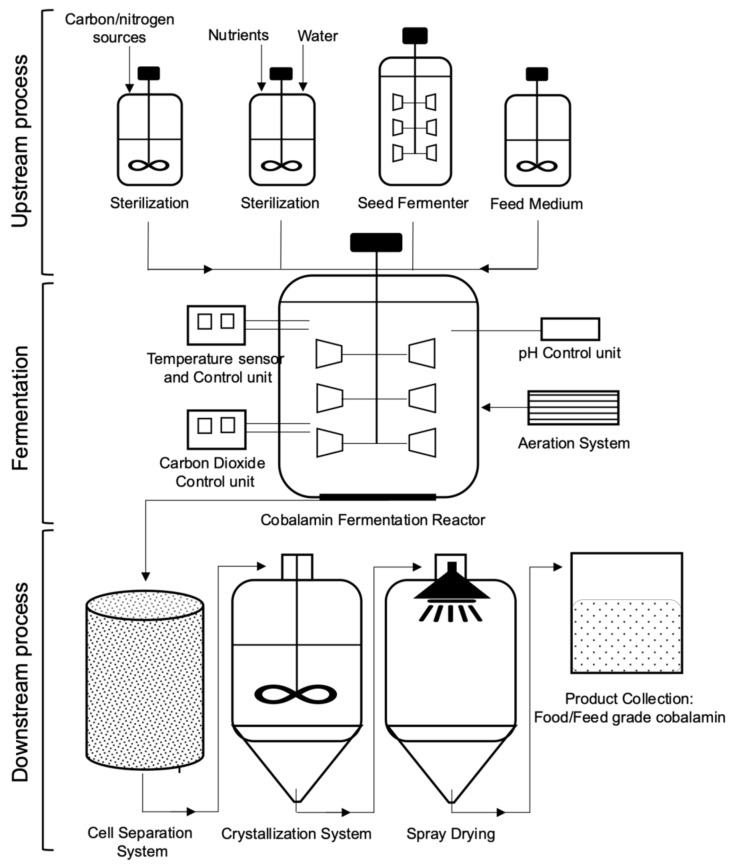
General flowsheet of the cobalamin industrial production applicable for aerobic process in *P. denitrificans*.

*P. denitrificans* is an industrial strain which is used by Sanofi Aventis (formerly Rhone Poulenc Rorer) for the large-scale production (Figure 5). The studies on B12 production were carried out with the strain *P. denitrificans* MB580 to obtain the overproducing derivative strain SC510 [54]. The titers of Cbl obtained with *P. denitrificans* are similar to *Propionobacterium* strains yielded about 300 mg/L in aerobic conditions [54]. The elucidation of Cbl biosynthesis and further amplification of eight genes from the *cobF-cobM* operon resulted in increase of the production in 30%. Later, the Cbl yield was increased (20%) via the copies of *cob A* and *cobE* genes [25,101].

The industrial process of vitamin B12 using *P. denitrificans* occurs at 30 °C and pH values at 6.0–7.0 in 120 m^3^ fermenters, using sucrose as carbon source and nitrogen sources as yeast extract in the presence of mineral salts, and lasts about 6–7 days [9]. The fermentation process is aerated during the growth phase and controlling by the dissolved oxygen and CO_2_ concentrations giving the Cbl yield more 150 mg/L (Figure 5). Later, the industrial Cbl synthesis was improved via multi-stage dissolved oxygen concentration (DOC) control strategy, which allowed enhancing the vitamin B12 yield by about 20% (70 mg/L). The fermentation started with a high level of dissolved oxygen concentration (8–10%) followed by its reduction to 2–5% (49–106 h) and further below 2% (107–168 h) [101,116]. Further improvement of the optimal CO_2_ fraction control strategy showed that the defined mixture of air and CO_2_ led to increasing of the vitamin B12 production by about 10% [117].

The influence of essential compounds of the medium on the aerobic Cbl biotechnology has been extensively studied too. At the beginning of the culture growth, the medium has to be supplemented with 10–25 mg/L of DMB and 40–200 mg/L Co^2+^ nitrate. The action of betaine and choline as methyl donors was shown to stimulate the ALA precursor, resulting in the increased B12 production [54]. Glutamate, glycine and methionine as the ALA precursors were also stimulated by betaine. Sugar beets molasses, which are the preferable carbon sources, contains the high betaine and glutamate amounts. The influence of betaine during the Cbl production process was beneficial when continuous feeding betaine to maintain its concentration in the broth in a range of 5–7 g/L during 50–140 h in the 120-m^3^ fermenter, resulting about 10% of B12 [101]. Currently, under the optimal fermentation conditions, the vitamin B12 is accumulated in *P. denitrificans* with the highest yield of 214.3 mg L^−1^ [101,105].

After the fermentation, a mixture of AdoCbl, MeCbl and OHCbl is subjected to chemical processes, including cyanidation, followed by extraction and purification steps with the use of organic solvents. Usually, the whole broth or an aqueous suspension of the harvested cells is heated at 80–120 °C for 10–30 min at pH 6.5–8.5 in order to extract the vitamin B12. The conversion to CNCbl is obtained by treating the heated broth or cell suspension with potassium cyanide or thiocyanate [54]. After clarification of the whole solution, via filtration or treatment with zinc hydroxide, the vitamin B12 is precipitated by the addition of auxiliaries like tannic acid or cresol. This procedure leads to a product of about 80% purity, which is used as animal feed additive. However, further purification via the different extraction steps by organic solvents (cresol, carbon tetrachloride and water/butanol) is often supplemented through the adsorption to ion exchangers or activated carbon [9]. Finally, the vitamin B12 is crystallized by the addition of organic solvents, leading to a product of recommended quality for food and pharmaceutical applications (Figure 5).

The current B12 bioprocess is yet suboptimal: it involves long fermentation cycles, expensive media and lack of genetic tools for commercial hosts. To develop efficient bioprocesses, genetic tools must be available and the host must be suitable for fermentation. The additional problems are the different water-solubility of the B group vitamins at the last stage of co-production and using potassium cyanide, which is not sustainable. The chemical conversion step into CNCbl and any subsequent purification steps cause this production process to be expensive, unsafe to the operators and environmentally unfriendly. In addition, MeCbl and CNCbl were found to effect on the gut microbiome and microbial metabolism differently [11]. MeCbl more effectively stimulate of the microbial lipid, terpenoid, and polyketide metabolism, as well as their utilization of exogenous substances. Thus, for future industrial process it is necessary to search new direction in optimization of strains, media, or process parameters, with using recycling materials to decrease the impact on environment.

## 8. Conclusions

Each ecosystem in Nature possesses a balanced content of inhabitants, which have evolved different metabolic capability and interdependence, including different levels of auxothrophy and protothrophy towards the essensial and chemically complex B12 coenzyme family cofactors. The major producers of the B12 family cofactors in Nature were found to be Proteobacteria, Actinobacteria, Firmicutes, Nitrospirae, and Thaumarchaeota in soil; Proteobacteria, Thaumarchaeota and Cyanobacteria (mostly pseudocobalamin) in the ocean, and Fusobacteria, Actinobacteria, Proteobacteria, Bacteroidetes, and Firmicutes among the gut microbial community. The most of these bioinformatically predicted B12-producers need experimental verification of their ability to synthesize cobalamin at the transcriptomic and biochemical levels. Microorganisms have applied the different evolutionary strategies, from the partial biosynthesis and transport of a cobamide, with the use of different exogenous precursors, to intraspecies differentiation of metabolic labour between the *de novo* B12-producing cells, continuously changing their lyfe style in dependence on the environment. However, the largest part (up to 80%) of each microbial community are the B12 auxotrophs of the different levels (Cba, Cbi, etc.). Therefore, it is important to support the natural producers of cobalamin or deliver it by supplementation in both eukaryotic and prokaryotic ecosystems. The analyses of microbial communities’ profiles and their genetic potentiality together with the tools of metabolic engineering should be possible to generate novel strains of interest with the high yield of vitamin B12. A promising and inexpensive method is the fermentation of grain and dairy products with propionobacteria and lactobacilli safe for humans and animals. However, the soil resources of the microbial cobalamin producers make it possible to develop an effective and safe method for enriching plant foods and mushrooms with vitamin B12 for vegetarians.

To date, the industrial production of vitamin B12 occurs via microbial synthesis mainly by the producers *P. denitrificans* (214 mg/L) and *P. freudenreichii* (206 mg/L), but their strains grow slowly (fermentation cycle is about 180 h) and have complicated ways for genetic manipulations. Thus, the current production of vitamin B12 should be improved via the introduction of new producing hosts. Over the last 10 years accumulated knowledge of the vitamin B12 biosynthetic steps and their regulations have helped engineer the de novo biosynthesis of vitamin B12 in well-studied and industrially suitable species such as *E. coli, B. megaterium*, and the soil species *S. meliloti*, which can be the alternative high-yield producing strains, giving significant engineering challenges for future studies. Successful demonstrations of the engineered strains for vitamin B12 production for short period of time (24–48 h), usage of non-expensive media components for fermentation, and directed gene manipulations allow providing the scientists with new considerations on improvement of B12 biosynthesis. Althougth the lists of microbial and genetic resources are continuously extended, the recombinant biosynthetic and regulatory genes (promoters and riboswitches) of only a few species such as *P. freudenreichii*, *P. denitrificans, R. capsulatus, B. melitensis, S. meliloti, S. typhimurium*, and *Rhodopseudomonas palustris* have been applied. Modern methods of comparative genomics and metabolic reconstruction will make it possible to discover promising hosts and, possibly, genes, regulatory elements or pathways for the more effective metabolism of cobalamin or the concerned and concomitant pathways. Moreover, there are still environmental and regulatory issues within the vitamin B12 process. Meanwhile, whereas vitamin B12 is produced by fermentation, the conversation of AdoCbl into air-stable CNCbl is exclusively a chemical process, which is not suitable, and therefore the findings of the replacement of this step biologically could result in a greener bioprocess.

## Figures and Tables

**Table 1:** Microbial genomes containing *cobU/T* orthologues with predicted ArsAB function for phenolyl Cba synthesis.

Species/Strain *	Isolation Source **	GeneBank Accession **
*Acetonema longum* APO-1 DSM 6540	gut, termite, *Pterotermes occidentis*	AFGF00000000
*Anaeroarcus burkinensis* DSM 6283	rice field soil	AUMI00000000
*Anaeromusa acidaminophila* DSM 3853	anaerobic purification plant	ARGA00000000
*Dendrosporobacter quercicolus* DSM 1736	discolored tissue in living oak tree	FNHB00000000
*Dialister succinatiphilus* YIT 11850	human faeces	CABKRA000000000
*Pelobacter seleniigenes* DSM 18267	freshwater wetland system	QKBM00000000
*Pelosinus fermentans* R7 DSM 17108	kaolin deposit Zhuravlinii Log	DJJR00000000
*Pelosinus propionicus* DSM 13327	gut of the termite Thoracotermes macrothorax Sjöstedt	FOTS00000000
*Sporomusa acidovorans* DSM 3132	effluent from alcohol-distillery plant	FNAM00000000
*Sporomusa malonica* DSM 5090	freshwater mud	FWXI00000000
*Veillonella atypica* KON ATCC 17744	human mouth	CP020566
*Veillonella montpellierensis* DSM 17217	human, gastric fluid of a newborn	AUFY00000000
Veillonella parvula *** DSM 2008	intestinal tract	CP001820
*Veillonella tobetsuensis* ATCC BAA-2400	tongue biofilm of healthy human adult	BBXI00000000
Sporomusa ovate *** DSM 2662	sugar beet leaf silage	ASXP00000000

*—According to metabolic reconstruction [7]; **—according to the BacDive database [50]; ***—experimentally confirmed phenolyl Cba synthesis [46,47,48,49].

## Appendix A

**Table A1:** Distribution of cobalamin/B12-related pathways in human gut microbial genomes according to Rodionov et al. [12].

Species	Genome ID */ Numbers **	Target Genes (Basic Pathway Variant)	*** Prototroph(P)/ Auxotroph(A) and Salvager(S)
*Eubacteriaceae bacterium* *Leptotrichia buccalis* *Leptotrichia goodfellowii* *Fusobacterium mortiferum* *Peptostreptococcus anaerobius* *Clostridium tyrobutyricum*	796939.3/4 523794.5/1 596323.3/1 469616.3/1 1122950.3/3 1121342.3/2	CbiK/CbiX/CbiX2, CbiL, CbiH, CbiF, CbiG, CbiD, CbiJ, CbiT, CbiE, CbiC, CbiA, Co-transporter, CbiP, CbiB, CobU, CobS, CobC/CblZ, CobT, CobD, BtuR/PduO	**P1** 628 genomes (28.2%)
*Clostridium hathewayi* *Lachnospiraceae bacterium* *Peptostreptococcus anaerobius* *Bilophila wadsworthia* *Bacteroides cellulosilyticus* *Bacteroides dorei* *Bacteroides fragilis* *Bacteroides intestinalis* *Bacteroides pyogenes* *Bacteroides stercoris* *Bacteroides uniformis* *Bacteroides vulgatus* *Parabacteroides distasonis* *Parabacteroides goldsteinii* *Parabacteroides johnsonii* *Parabacteroides merdae* *Porphyromonas asaccharolytica* *Porphyromonas gingivalis* *Fusobacterium gonidiaformans* *Fusobacterium necrophorum* *Fusobacterium nucleatum* *Fusobacterium periodonticum* *Citrobacter amalonaticus* *Citrobacter freundii* *Citrobacter koseri* *Klebsiella oxytoca* *Klebsiella pneumoniae* *Salmonella enterica (Agona)* *Salmonella enterica (Typhum)* *Salmonella enterica* *Edwardsiella tarda* *Hafnia alvei* *Morganella morganii* *Yersinia enterocolitica* *Yersinia frederiksenii* *Yersinia kristensenii* *Desulfovibrio desulfuricans* *Propionibacterium acidipropionici* *Propionibacterium acnes* *Propionibacterium granulosum* *Propionibacterium freudenreichii* *Propionibacterium subsp. Shermanii* *Bacillus megaterium* *Lysinibacillus fusiformis* *Lysinibacillus sphaericus* *Listeria innocua* *Listeria monocytogenes* *Brevibacillus brevis* *Lactobacillus reuteri* *Streptococcus sanguinis* *Clostridium acetobutylicum* *Clostridium botulinum* *Clostridium perfringens* *Ruminococcus obeum* *Blautia wexlerae* *Dorea formicigenerans* *Eubacterium saburreum*	566550.8/3 658081.3/8 596329.3/3 563192.3/1 246787.4/3 997875.3/4 272559.17/6 329854.7/2 1321819.3/3 449673.7/2 411479.10/6 435590.9/5 435591.13/3 927665.4/3 537006.5/2 411477.4/3 879243.3/2 431947.6/4 469615.3/2 1095747.3/3 525283.3/12 546275.3/3 35703.30/4 1006003.3/7 290338.8/3 1191061.3/19 1037908.3/9 1265617.3/2 99287.12/6 550537.5/7 500638.3/6 1002364.3/4 582.34/4 630.33/7 349966.8/2 28152.3/2 525146.4/3 1748.13/3 1091045.3/83 1160719.7/2 66712.6/2 754252.3/1 1348623.7/6 1231627.3/2 1421.39/6 1002366.3/2 882095.3/12 1129895.3/2 557436.4/7 997356.4/20 272562.8/2 441770.6/7 195103.9/4 411459.7/2 1121115.4/2 411461.4/2 887325.3/2	CbiK/CbiX/CbiX2, CbiL, CbiH, CbiF, CbiG, CbiD, CbiJ, CbiT, CbiE, CbiC, CbiA, Co-transporter, CbiP, CbiB, CobU, CobS, CobC/CblZ, CobT, CobD, BtuR/PduO; +transporters: CbrUVT/BtuBCDF	**P1&S**
*Lachnobacterium bovis* *Clostridium bolteae* *Clostridium clostridioforme* *Clostridium symbiosum* *Roseburia intestinalis* *Eubacterium rectale* *Desulfitobacterium hafniense* *Clostridium difficile* *Clostridium sordellii* *Clostridium bifermentans* *Faecalibacterium prausnitzii* *Selenomonas ruminantium* *Veillonella atypica* *Veillonella parvula*	140626.5/3 997893.5/6 999403.4/9 411472.5/3 536231.5/3 515619.6/3 272564.6/4 499175.4/17 1292036.3/2 1233170.3/3 411483.3/4 1280706.4/2 866776.4/3 686660.3/3	ChlID, CobN, CbiL, CobG, CbiH, CbiF, CobF, CbiJ, CbiT, CbiE, CbiC, CbiA, Co-transporter, CbiP, CbiB, CobU, CobS, CobC/CblZ, CobT, CobD, BtuR/PduO	**P2** 97 genomes (4,4%)
*Mesorhizobium loti* *Agrobacterium tumefaciens* *Corynebacterium glucuronolyticum* *Corynebacterium kroppenstedtii* *Corynebacterium ulcerans* *Gordonia terrae* *Mycobacterium avium* *Mycobacterium abscessus* *Bradyrhizobium japonicum* *Ochrobactrum anthropi* *Burkholderia cepacia* *Comamonas testosteroni* *Delftia acidovorans* *Pseudomonas aeruginosa* *Pseudomonas fluorescens* *Pseudomonas putida* *Dietzia cinnamea* *Rhodococcus equi* *Rhodococcus erythropolis* *Rhodococcus rhodochrous*	266835.9/2 176299.3/2 548478.3/2 645127.4/2 945711.3/2 1316928.3/2 243243.7/3 36809.5/3 224911.1/3 439375.7/3 292.26/3 1009852.3/3 883100.3/3 208964.1/12 294.123/11 303.200/13 1223524.3/2 525370.3/2 1833.80/4 1330040.3/2	+transporters: CbrUVT/BtuBCDF	**P2&S**
*Bacteroides sartorii* *Methyloversatilis universalis* *Pseudomonas stutzeri*	1236538.3/2 999628.3/2 32042.3/14	CbiA, CbiP, CbiB, CobU, CobS, CobC/CblZ, CobT, CobD, BtuR/PduO; +transporters: CbrUVT/BtuBCDF	**Aba&S**, 32 genomes (1.4%)
*Bacteroides eggerthii* *Prevotella buccae* *Akkermansia muciniphila* *Vibrio furnissii* *Vibrio parahaemolyticus* *Vibrio mimicus* *Paenibacillus sp.* *Paenisporosarcina sp.* *Turicibacter sp.*	665953.3/2 873513.3/2 349741.6/3 675811.4/2 670.436/4 674.46/3 1033743.3/6 1078085.3/2 702450.3/2	CbiP, CbiB, CobU, CobS, CobC/CblZ, CobT, CobD, BtuR/PduO; +transporters: CbrUVT/BtuBCDF	**Acbr&S**, 43 genomes (1.9%)
*Rudanella lutea* *Nevskia ramosa* *Arcobacter butzleri* *Bacteroides ovatus* *Prevotella oralis* *Myroides odoratimimus* *Escherichia albertii* *Escherichia coli* *Enterobacter aerogenes* *Shigella dysenteriae* *Shigella flexneri*	1089547.3/1 1122603.3/1 888827.3/1 411476.11/6 873533.3/2 76832.7/6 208962.36/3 481805.6/88 548.155/5 622.8/3 623.158/5	CobU, CobS, CobC/CblZ, CobT, CobD, BtuR/PduO	**Acbi** 193 genomes (8.7%)
*Shigella sonnei* *Acinetobacter baumannii* *Acinetobacter calcoaceticus* *Acinetobacter pittii* *Acinetobacter haemolyticus* *Acinetobacter johnsonii* *Acinetobacter junii* *Acinetobacter radioresistens*	624.634/6 400667.7/19 981331.5/7 48296.83/4 707232.3/3 1217662.4/2 1217665.3/2 981334.6/4	+transporters: CbrUVT/BtuBCDF	**Acbi&S**, mostly E. coli and Acinetobacter spp.
*Lactobacillus brevis* *Lactobacillus crispatus* *Capnocytophaga ochracea* *Sutterella wadsworthensis* *Burkholderiales bacterium* *Neisseria flavescens* *Neisseria mucosa* *Actinobacillus pleuropneumoniae* *Haemophilus influenza* *Moraxella catarrhalis* *Campylobacter coli* *Campylobacter jejuni* *Campylobacter ureolyticus* *Helicobacter pylori* *Actinomyces odontolyticus* *Mobiluncus curtisii* *Mobiluncus mulieris* *Varibaculum cambriense* *Bifidobacterium adolescentis* *Bifidobacterium angulatum* *Bifidobacterium animalis* *Bifidobacterium bifidum* *Bifidobacterium breve* *Bifidobacterium dentium* *Bifidobacterium longum* *Micrococcus luteus* *Rothia mucilaginosa* *Cellulosimicrobium cellulans* *Gemella haemolysans* *Staphylococcus aureus* *Staphylococcus epidermidis* *Staphylococcus intermedius* *Staphylococcus lugdunensis* *Aerococcus viridans* *Enterococcus faecium* *Lactobacillus fermentum* *Lactobacillus iners* *Lactobacillus johnsonii* *Lactobacillus plantarum* *Lactobacillus ruminis* *Lactobacillus salivarius* *Pediococcus acidilactici* *Leuconostoc mesenteroides* *Weissella koreensis* *Lactococcus lactis* *Streptococcus intermedius* *Streptococcus parasanguinis* *Streptococcus pneumoniae* *Erysipelotrichaceae bacterium*	525310.3/2 679188.3/7 521097.5/3 742823.3/3 469610.4/2 546264.5/2 546266.6/2 1224142.3/5 727.532/11 480.212/2 195.462/6 683083.3/11 883165.3/3 85962.8/17 411466.7/3 887326.3/3 585199.3/4 184870.3/3 367928.6/3 518635.5/2 442563.4/11 500634.6/5 518634.7/22 871562.3/5 216816.111/21 1270.32/8 553201.3/4 1710.9/2 546270.5/2 1229492.3/42 176280.1/39 1141106.7/1 1034809.4/5 655812.3/2 1138892.3/24 525325.3/5 888801.3/14 525330.3/5 525338.3/7 525362.3/3 1041521.3/5 862514.3/4 203120.7/2 1045854.4/2 746361.3/6 1316583.3/6 1114965.3/4 561276.4/34 658659.3/6	BtuR/PduO	**A** 1235 genomes (55.4%)
*Anaerococcus prevotii* *Finegoldia magna* *Mycoplasma hominis* *Mycoplasma pneumoniae* *Ureaplasma parvum* *Ureaplasma urealyticum* *Lactobacillus reuteri* *Bacteroides caccae* *Bacteroides salyersiae* *Bacteroides thetaiotaomicron* *Bacteroides xylanisolvens* *Prevotella bivia* *Prevotella corporis* *Prevotella intermedia* *Prevotella melaninogenica* *Prevotella nigrescens* *Achromobacter xylosoxidans* *Alcaligenes faecalis* *Aeromonas caviae* *Aeromonas hydrophila* *Aeromonas media* *Aeromonas veronii* *Cronobacter sakazakii* *Enterobacter asburiae* *Enterobacter cloacae* *Enterobacter hormaechei* *Escherichia fergusonii* *Pantoea agglomerans* *Proteus mirabilis* *Proteus vulgaris* *Providencia alcalifaciens* *Providencia rettgeri* *Providencia stuartii* *Serratia marcescens* *Yersinia pseudotuberculosis* *Plesiomonas shigelloides* *Aggregatibacter aphrophilus* *Haemophilus parainfluenzae* *Stenotrophomonas maltophilia* *Brachyspira pilosicoli* *Tropheryma whipplei* *Eggerthella lenta* *Bacillus cereus* *Bacillus mycoides* *Bacillus thuringiensis* *Bacillus amyloliquefaciens* *Bacillus licheniformis* *Bacillus subtilis* *Enterococcus faecalis* *Enterococcus casseliflavus* *Lactobacillus acidophilus* *Lactobacillus amylovorus* *Lactobacillus casei* *Lactobacillus delbrueckii* *Lactobacillus gasseri* *Lactobacillus helveticus* *Lactobacillus rhamnosus* *Lactococcus garvieae* *Streptococcus agalactiae* *Streptococcus infantis* *Streptococcus mitis* *Streptococcus mutans* *Streptococcus oralis* *Streptococcus parauberis* *Streptococcus pyogenes* *Streptococcus thermophilus* *Eubacterium siraeum* *Ruminococcus albus*	879305.3/2 334413.6/5 1267000.3/2 272634.6/4 515608.4/5 565575.4/14 349123.6/3 411901.7/2 997887.3/2 226186.1/4 657309.4/2 868129.3/2 28128.5/2 28131.5/2 553174.6/2 702439.3/2 762376.5/2 511.9/2 648.77/3 644.34/4 651.7/3 654.45/5 290339.8/5 61645.42/3 716541.4/6 1259823.3/3 550694.3/3 549.25/7 525369.4/4 585.10/2 520999.6/2 521000.6/2 471874.6/4 615.109/5 349747.9/4 703.7/2 985008.3/4 888828.3/5 40324.126/4 1133568.3/2 203267.6/2 479437.5/2 1396.420/35 526997.3/4 527031.3/26 692420.6/10 279010.13/4 1423.136/11 525271.4/62 565654.4/4 1314884.3/4 695560.3/2 321967.11/6 321956.7/4 324831.13/7 585520.4/4 1088720.3/6 420889.6/3 1309806.3/16 889204.3/4 864567.3/12 1257041.3/9 1161421.3/9 936154.3/5 864568.3/9 1051074.3/6 428128.7/3 246199.4/1	BtuR/PduO; +transporters: CbrUVT/BtuBCDF	**A&S**

*—an example of a species genome is presented; **—the numbers of genomes of a species with the pathway variants: ***—**P1** and **P2**-*de novo* anaerobic and aerobic Cbl biosynthesis (protothrophy), respectively; **P1&S** and **P2&S**-*de novo* anaerobic and aerobic biosynthesis and salvage; **Aca&S**-cobyrinate auxotrophy and salvage; **Acbr&S**-cobyrinate diamide auxotrophy and salvage; **Acbi&S**-cobinamide auxotrophy and salvage; **A&S**-cobalamin/B12 auxotrophy and salvage).

**Table A2:** Actively cobamide-producing microorganisms with the identified cobamide biosynthesis genes and pathways according to Shelton et al. [7].

Species	Biosynthesis Pathway	Classification
*Streptomyces coelicolor*	aerobic	Actinobacteria
*Streptomyces griseus*		
*Agrobacterium tumefaciens* *Methylobacterium extorquens* *M. dichloromethanicum* *Methylosinus trichosporium* *Sinorhizobium meliloti*	aerobic	α-Proteobacteria Rhizobiales
*Dinoroseobacter shibae* *Rhodobacter capsulatus* *Rhodobacter sphaeroides* *Ruegeria pomeryoi*	aerobic	α-Proteobacteria Rhodobacterales
*Rhodospirillum rubrum*	aerobic	α-Proteobacteria Rhodospirillales
*Methylobacter luteus* *Methylococcus capsulatus*	aerobic	γ-Proteobacteria Methylococcales
*Pseudomonas denitrificans* *Pseudomonas putida*	aerobic	γ-Proteobacteria Pseudomonadales
*Propionibacterium acidipropionici* *Propionibacterium freudenreichii* *Propionibacterium shermanii*	anaerobic	Actinobacteria
*Prochlorococcus sp. MIT9313* *Synechococcus elongates* *Synechocystis sp. PCC6803*	anaerobic	Cyanobacteria
*Bacillus megaterium* *Listeria monocytogenes*	anaerobic	Firmicutes Bacilli Bacillales
*Lactobacillus coryniformis* *Lactobacillus reuteri* *Lactobacillus rossiae*	anaerobic	Firmicutes Bacilli Lactobacillales
*Clostridium cochlearium* *Clostridium kluyveri*	anaerobic	Firmicutes Clostridia Clostridiales Clostridiaceae
*Acetobacterium woodii* *Eubacterium barkeri* *Eubacterium hallii* *Eubacterium limosum*	anaerobic	Firmicutes Clostridia Clostridiales Eubacteriaceae
*Blautia hydrogenotrophica* *Clostridium phytofermentans*	anaerobic	Firmicutes Clostridia Clostridiales Lachnospiraceae
*Dehalobacter restrictus* *Desulfitobacterium hafniense* *Desulfitobacterium sp. PCE1* *Desulfotomaculum reducens*	anaerobic	Firmicutes Clostridia Clostridiales Peptococcaceae
*Moorella thermoacetica*	anaerobic	Firmicutes Clostridia Thermoanaerobacterale
*Pelosinus fermentans* *Sporomusa ovata* *Veillonella parvula*	anaerobic	Firmicutes Negativicutes
*Desulfobacterium autotrophicum* *Desulfobulbus propionicus*	anaerobic	δ-Proteobacteria Desulfobacterales
*Desulfovibrio desulfuricans* *Desulfovibrio vulgaris*	anaerobic	δ-Proteobacteria Desulfovibrionales
*Geobacter lovleyi* *Geobacter sulfurreducens* *Pelobacter propionicus*	anaerobic	δ-Proteobacteria Desulfuromonadales
*Sulfurospirillum multivorans*	anaerobic	ε-Proteobacteria
*Salmonella typhimurium* *Yersinia enterocolitica*	anaerobic	γ-Proteobacteria
*Thermosipho africanus H1760334* *Thermosipho africanus TCF52B*	anaerobic	Thermotogae
*Aphanizomenon flos-aquae* *Crocosphaera watsonii*	unknown	Cyanobacteria
*Clostridium tetanomorphum*	unknown	Firmicutes
